# Genetic reduction of mTOR extends lifespan in a mouse model of Hutchinson‐Gilford Progeria syndrome

**DOI:** 10.1111/acel.13457

**Published:** 2021-08-28

**Authors:** Wayne A. Cabral, Urraca L. Tavarez, Indeevar Beeram, Diana Yeritsyan, Yoseph D. Boku, Michael A. Eckhaus, Ara Nazarian, Michael R. Erdos, Francis S. Collins

**Affiliations:** ^1^ Molecular Genetics Section Center for Precision Health Research National Human Genome Research Institute National Institutes of Health Bethesda MD USA; ^2^ Translational Musculoskeletal Innovation Initiative Carl J. Shapiro Department of Orthopedic Surgery Beth Israel Deaconess Medical Center Harvard Medical School Boston MA USA; ^3^ Diagnostic and Research Services Branch Division of Veterinary Resources Office of the Director National Institutes of Health Bethesda MD USA

**Keywords:** lamin A/C, laminopathies, mTOR, progeria, S6 Kinase

## Abstract

Hutchinson‐Gilford progeria syndrome (HGPS) is a rare accelerated aging disorder most notably characterized by cardiovascular disease and premature death from myocardial infarction or stroke. The majority of cases are caused by a de novo single nucleotide mutation in the *LMNA* gene that activates a cryptic splice donor site, resulting in production of a toxic form of lamin A with a 50 amino acid internal deletion, termed progerin. We previously reported the generation of a transgenic murine model of progeria carrying a human BAC harboring the common mutation, G608G, which in the single‐copy state develops features of HGPS that are limited to the vascular system. Here, we report the phenotype of mice bred to carry two copies of the BAC, which more completely recapitulate the phenotypic features of HGPS in skin, adipose, skeletal, and vascular tissues. We further show that genetic reduction of the mechanistic target of rapamycin (mTOR) significantly extends lifespan in these mice, providing a rationale for pharmacologic inhibition of the mTOR pathway in the treatment of HGPS.

## Graphical abstract

Generation of animal models that express the mutant gene product in the context of the human sequence is critical for developing novel therapeutic approaches to treat HGPS. Here we show that transgenic mice harboring two copies of the human *LMNA* gene containing the G608G mutation recapitulate the human phenotype. Genetic reduction of mTOR in these mice extends lifespan and provides a rationale for clinical trials testing pharmacological inhibition of the mTOR signaling pathway in HGPS patients.

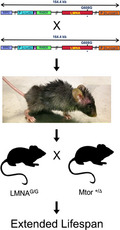

**Abbreviations**
ACTB: beta actin
BAC: bacterial artificial chromosome
BMD: bone mineral densirty
FTI: farnesyltransferase inhibitor
GAPDH: glyceraldehyde 3‐phosphate dehydrogenase
HGPS: Hutchinson‐Gilford progeria syndrome
IGF: insulin‐like growth factor
LMNA: lamin A
LMNC: lamin C
mTOR: mechanistic target of rapamycin
PCR: polymerase chain reaction
SMA: smooth muscle actin
VSMC: vascular smooth muscle cell
μCT: micro‐Computed Tomography

## 1 INTRODUCTION

Hutchinson‐Gilford progeria syndrome (HGPS) is a fatal autosomal dominant segmental premature aging disorder that occurs in approximately 1 in 4–8 million births (Hennekam, 2006). Progressively developing features of HGPS include growth deficiency, alopecia, loss of subdermal fat, sclerodermatous skin, and musculoskeletal abnormalities including relative mandibular and clavicular hypoplasia, joint contractures, and osteoporosis (Ullrich & Gordon, 2015). The most serious aspects of the disease involve loss of vascular smooth muscle cells (VSMC) in the medial layer of large arteries which are replaced by proteoglycan‐rich extracellular matrix and development of generalized features of atherosclerosis with focal areas of calcification (Gerhard‐Herman et al., 2012). In the majority of HGPS cases, death occurs as a result of complications of severe cardiac disease (myocardial infarction or heart failure) or cerebrovascular disease (stroke) at an average age of 14.6 years.

Classic HGPS is caused by a *de novo* mutation (c.1824C→T, p.G608G) in the *LMNA* gene, encoding the A‐type lamins A, C, and AΔ10 (Eriksson et al., 2003; De Sandre‐Giovannoli et al., 2003). Along with B‐type lamins, A‐type lamins comprise the nuclear lamina underlying the inner nuclear membrane. Prelamin A is subject to several post‐translational processing events that include farnesylation of the cysteine in the C‐terminal CAAX sequence, cleavage of the terminal AAX sequence with addition of a methoxy group to the terminal cysteine, and subsequent cleavage of the terminal 15 amino acid residues by the metalloprotease ZMPSTE24. Although the G608G mutation does not alter the *LMNA* coding sequence, activation of an exonic cryptic splice donor site produces an in‐frame protein with an internal deletion, termed progerin, that excludes the ZMPSTE24 cleavage recognition site. Permanently farnesylated progerin functions in a dominant‐negative manner to disrupt the roles of the nuclear lamina in regulating nuclear shape, DNA replication, transcription, cell division, and chromatin organization (Kubben & Misteli, 2017).

Development of a transgenic model that reproduces the human HGPS phenotype should be useful in testing therapeutic approaches to target either the mutation or mutant gene product in the context of the human sequence. We previously reported the creation of a transgenic mouse harboring the G608G mutated human *LMNA* on a 164‐kb bacterial artificial chromosome (BAC), designated as C57BL/6‐Tg(LMNA*G608G)HClns/J, and henceforth referred to as *LMNA* ^G/+^ (Varga et al., 2006). Although the *LMNA* transgene expresses human lamin A, lamin C, and progerin in all tissues tested, mice carrying a single copy of the human BAC (*LMNA* ^G/+^) exhibit normal growth and external phenotypic features compared to wild‐type littermates or mice carrying a normal human *LMNA* transgene. The most dramatic finding was the progressive loss of VSMC, particularly in descending aorta and carotid artery, that was associated with thickening of the adventitia and medial layer, and proteoglycan accumulation by 5 months of age. Arterial calcification was observed in older mice with severe VSMC loss and extracellular matrix deposition. Thus, while single‐copy transgenic mice (*LMNA* ^G/+^) lack the external phenotype seen in patients, this model demonstrates the progressive vascular abnormalities that closely resemble the most lethal aspect of HGPS (Varga et al., 2006).

Aging‐related disorders such as cancers, diabetes, cardiovascular, and neurodegenerative diseases, as well as normal aging, have been linked to dysregulated signaling of the mechanistic target of rapamycin (mTOR) pathway (Dazert & Hall, 2011). While genetic reduction of mTOR, mLST8, or S6K1 in mice increases lifespan and inhibits the onset of aging‐related diseases, inhibitors of mTOR signaling components have drawn interest as potential treatments for disorders associated with deposition of insoluble protein aggregates by means of enhanced clearance through autophagic‐lysosomal pathway activation (Selman et al., 2009; Wu et al., 2013). In cell culture experiments, treatment with rapamycin, or its analogs everolimus and temsirolimus, has been demonstrated to reduce progerin aggregates and nuclear blebbing, enhance cell proliferation, and delay the onset of cellular senescence, thereby providing evidence that chemical inhibition of mTOR signaling might restore cellular homeostasis in HGPS (Cao et al., 2011; Cenni et al., 2011; DuBose et al., 2018). These findings have led to a clinical trial of everolimus in children with HGPS, with results expected within a year. Here, we present the phenotypic characterization of double‐copy *LMNA* G608G transgenic mice, which faithfully recapitulate vascular, dermal, adipose, and skeletal features that develop in HGPS patients. Furthermore, we report the beneficial effects of genetic mTOR reduction in these mice as a rationale for pharmacologic intervention in HGPS therapy.

## 2 RESULTS

We have previously developed a transgenic mouse model of HGPS by using a 164Kb circular BAC containing 4 functional genes (*UBQLN4*, *RAB25*, *MEX3A*, and a recombineered *LMNA*) in addition to incomplete sequences of two additional genes, *SSR2* and *SEMA4A (*Varga et al., 2006 *)*. Initial efforts to characterize the structure of the transgene expressed in our mouse model utilized hybridization capture and short‐read NGS sequencing. Although this analysis was useful for obtaining the genomic location of the insertion sites on murine chromosome 4, it lacked the resolution required to resolve the actual transgene configuration and any recombination events that occurred during integration (Dubose et al., 2013). We therefore employed whole‐genome sequencing of transgenic mouse DNA and comparison analyses with BAC sequence to determine the precise structure and contents (Figure S1a). Fine mapping of the transgene identified three separate copies of human *LMNA*, the first of which includes sequence covering exon 1 and portions of the first intron, a second copy that includes exons 2 −12, and a third copy consisting of the full gene sequence with the G608G mutation. Partial gene sequences were identified for *SSR2* and *SEMA4A*, as expected. A third gene, *MEX3A*, localized to an inversion junction and underwent multiple recombination events resulting in several fragments averaging ~400–500 bp each. Since *UBQLN4* sequence was entirely absent from WGS reads we concluded that only two functional genes are encoded within the transgene, including the mutant *LMNA* and normal *RAB25* genes.

Similar to HGPS patients, mice carrying either one (*LMNA* ^G/+^) or two copies (*LMNA* ^G/G^) of the human *LMNA* G608G transgene are indistinguishable from wild‐type littermates at birth. We noted, however, that the genotype distribution of offspring generated by matings between mice harboring one copy of the transgene slightly deviated from the predicted Mendelian ratio, with no observed lethality in *LMNA* ^G/+^ mice but 12% lethality in *LMNA* ^G/G^ (Figure S1b). Although no difference was observed between male and female mice (n = 150 per gender), these numbers suggest a moderate level of either embryonic or perinatal lethality in *LMNA* ^G/G^ offspring.

**FIGURE 1:**
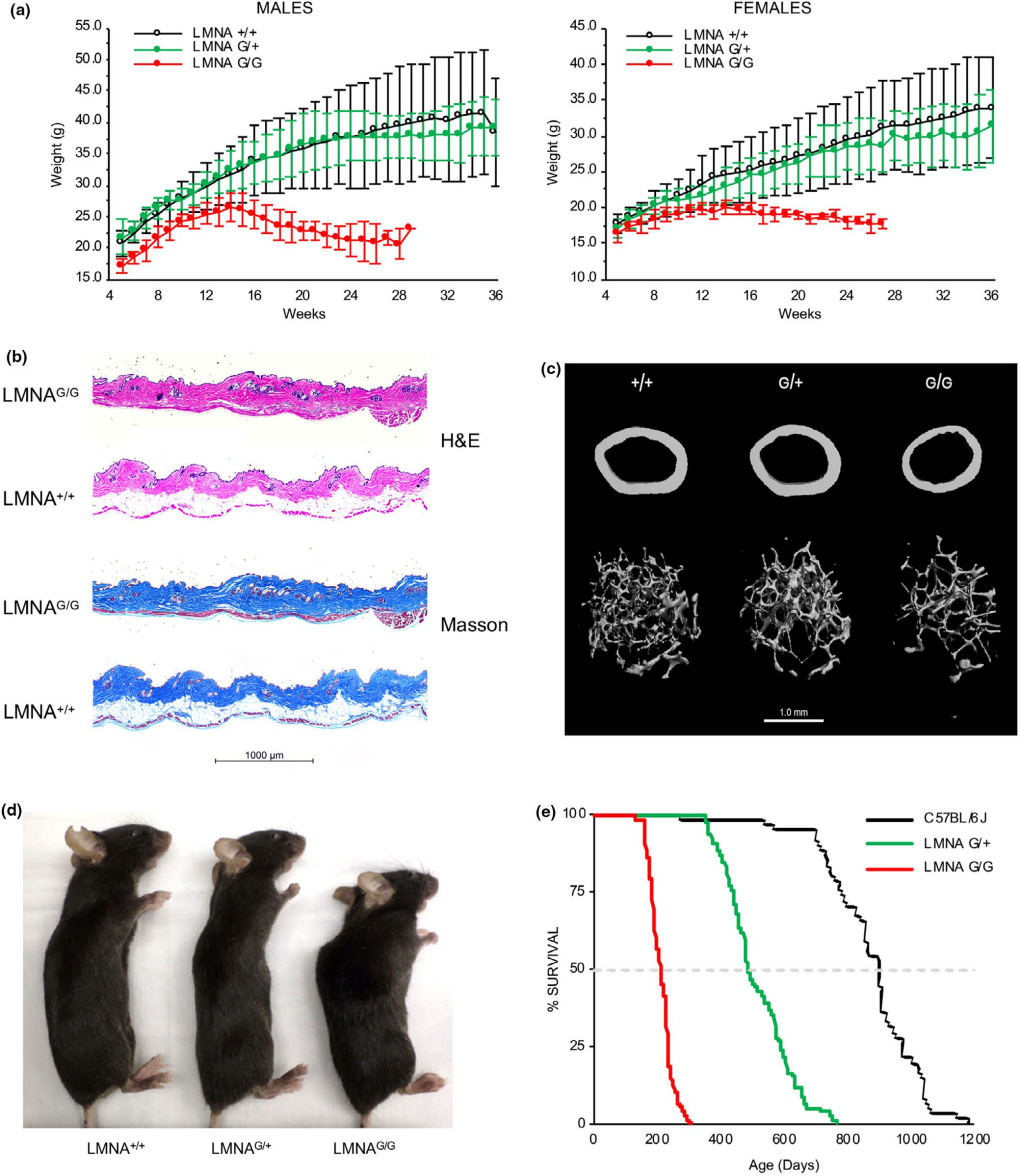
Murine model harboring two copies of the human *LMNA* G608G transgene develops a severe progeroid phenotype. (a) Growth curves of wild‐type (*LMNA* ^+/+^; n = 16 males, 13 females), mice carrying one copy (*LMNA* ^G/+^; n = 8 males, 8 females), or two copies (*LMNA* ^G/G^; n = 9 males, 8 females) of the human transgene containing the *LMNA* G608G mutation. By 5 weeks of age, *LMNA* ^G/G^ weigh significantly less than *LMNA* ^+/+^ and *LMNA* ^G/+^ littermates. Males, 5–15 weeks *p *< 0.01, 16–28 weeks *p *< 0.001; Females, 5–15 weeks *p *< 0.05, 16–23 weeks *p *< 0.01, 24–27 weeks *p *< 0.001. (b) Histologic analysis of skin from *LMNA* ^G/G^ and wild‐type (*LMNA* ^+/+^) littermates at 6 months of age demonstrates decreased subcutaneous fat in transgenic mice. Skin sections were stained with hematoxylin and eosin (H&E) and Masson's trichrome, which reveals the keratin and muscle (red), collagen (blue), and cellular cytoplasm (pink) and nuclei (dark brown). The unstained adipose tissue is located between the collagenous dermal layers (blue) and dark red‐stained panniculus carnosus (fast twitch type IIB glycolytic fibers). (c) Micro‐computed tomography (μCT)‐derived images of femoral bone of wild‐type (+/+), single‐copy (G/+) and double‐copy (G/G) transgenic mice at 6 months of age. Both cortical (top images) and trabecular (bottom images) structural parameters demonstrate the reduced bone volume in *LMNA* ^G/G^ mice. (d) Lateral images of 5‐month‐old mice reveal kyphosis and growth deficiency observed in double‐copy (*LMNA* ^G/G^) transgenic mice compared to wild‐type (*LMNA* ^+/+^) and single‐copy (*LMNA* ^G/+^) mice. (e) Kaplan‐Meier plots illustrating the shortened lifespan of single‐copy (*LMNA* ^G/+^, n = 79) and double‐copy (*LMNA* ^G/G^, n = 181) transgenic mice relative to wild type (*LMNA* ^+/+^; C57BL/6J, n = 61)

We previously reported that *LMNA* ^G/+^ transgenic mice lack pathologic changes outside of the vascular system prior to 20 months of age (Varga et al., 2006). In contrast to single‐copy mice, *LMNA* ^G/G^ mice developed phenotypic features consistent with HGPS following the first month after birth, relative to wild‐type (*LMNA* ^+/+^) littermates. By 5 weeks of age, both male and female *LMNA* ^G/G^ mice were growth deficient (Figure 1a). From 5 to 15 weeks of age, double‐copy transgenic mice weighed 10–20% less than *LMNA* ^+/+^ and *LMNA* ^G/+^ littermates. After 3.5–4 months of age, no additional weight gain was observed in *LMNA* ^G/G^ mice, with progressive wasting resulting in a 20–45% decrease in weight compared to *LMNA* ^+/+^ and *LMNA* ^G/+^ mice (*p *< 0.01 versus both genotypes) (Figure 1a, Figure S1c).

The decreased weight observed in *LMN*A^G/G^ mice relative to wild‐type (*LMNA* ^+/+^) and single‐copy transgenic (*LMNA* ^G/+^) littermates largely reflected growth deficiency, combined with the loss of adipose tissue and bone mass. Consistent with the lipodystrophic phenotype of HGPS patients, histopathologic analysis of skin from 6‐month‐old *LMNA* ^G/G^ mice demonstrated decreased dermal and subcutaneous adipose tissue resulting in tightened skin (Figure 1b). Although mild hair loss occurred infrequently in these mice, no obvious abnormalities of hair follicles were evident.

We also noted that *LMNA* ^G/G^, but not *LMNA* ^G/+^ mice, began to develop hindlimb contractures and moved with a waddling gait beginning at 6–7 months of age. To assess whether transgenic mice exhibited skeletal features similar to those observed in HGPS patients, bone morphology was evaluated in murine femurs at 6 months of age by μCT analysis (Figure 1c). All trabecular and cortical structural parameters in long bones of *LMNA* ^G/+^ mice were comparable to wild‐type (*Lmna* ^+/+^) littermates, reflecting the normal weights and the absence of pathologic features outside of the vascular system. In contrast, bone structural parameters were altered in *LMNA* ^G/G^ mice relative to wild type. Trabecular bone volume (BV/TV) was half of wild‐type values (*p *< 0.01), due to decreased trabecular thickness rather than a decrease in trabecular number, while cortical thickness (CT Th) was modestly reduced (~11%) in *LMNA* ^G/G^ femurs (*p *< 0.001). Additional musculoskeletal features observed in *LMNA* ^G/G^ mice included kyphosis, which developed by 4 months and progressed until mice reached endpoint (Figure 1d, Figure S1d).

**FIGURE 2:**
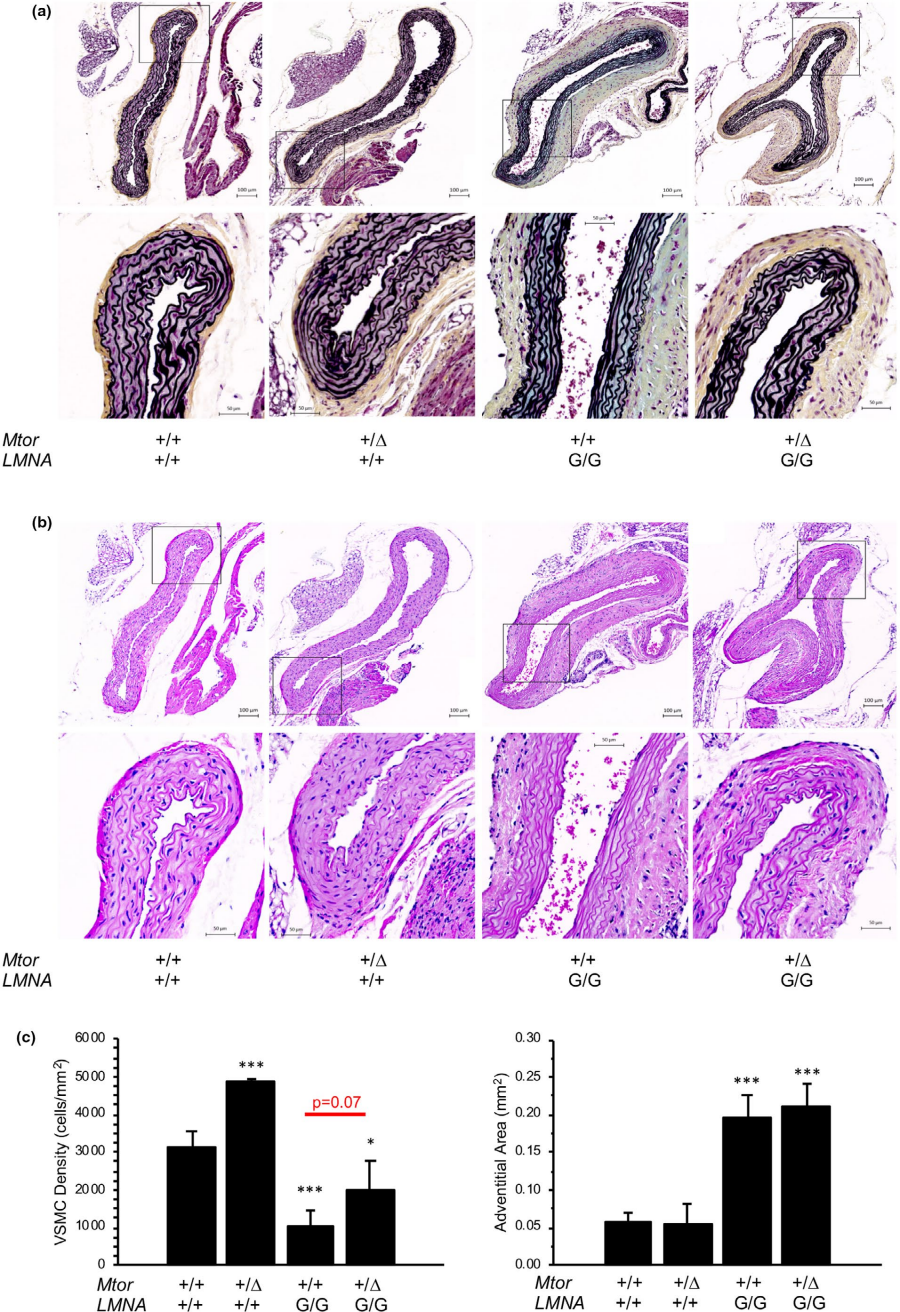
Severe vascular phenotype in HGPS mice can be partially rescued by genetic reduction of *Mtor*. (a) Movat's pentachrome‐stained ascending aorta sections from 5‐month‐old mice. (b) Hematoxylin and eosin (H&E)‐stained aortae from *LMNA* ^+/+^ and *LMNA* ^G/G^ mice. (c) Quantitation of adventitial area and VSMC number in aortae. Genetic reduction of *Mtor* partially restores the loss of vascular smooth muscle cells but does not rescue the adventitial expansion of aortae in *LMNA* ^G/G^ mice. *, *p *< 0.05; **, *p *< 0.01; ***, *p *< 0.001

Similar to *LMNA* ^G/+^ mice but even more extensive, progressive loss of VSMCs, elastic fiber breakage, proteoglycan, and collagen accumulation in the media, and dramatic thickening of the adventitia occurred in vascular tissues of 5‐month‐old *LMNA* ^G/G^ mice (Figure 2a–c). Arterial and arteriolar pathology was prominent in the aorta, but also included the carotid arteries with focal pathology in arteries and arterioles of the heart, kidneys, skeletal muscles, and adipose tissues. These vascular abnormalities were characterized by decreased numbers of smooth muscle nuclei, increased amounts of ground substance in the tunica media, and increased amounts of adventitial connective tissue. Adventitial expansion was associated with deposition of collagenous matrix, as noted by picrosirius red staining (Figure S1e).

Most notable, however, was the generalized severe phenotype exhibited in *LMNA* ^G/G^ mice, characterized by reduction of activity around 6 months of age and lasting until death by age 7–8 months (Figure 1e). The average lifespan of *LMNA* ^+/+^ and *LMNA* ^G/+^ mice was 897 and 485 days, respectively, compared to 212 days for *LMNA* ^G/G^ mice (p ~ 10^−137^ vs *LMNA* ^+/+^; p ~ 10^−101^ vs *LMNA* ^G/+^). Furthermore, there was no significant difference in average lifespan of male (216 ± 36 days) and female (208 ± 35 days) *LMNA* ^G/G^ mice. Despite the severe vascular alterations observed in *LMNA* ^G/G^ mice; however, post‐mortem analyses were unable to identify a specific acute cause of death.

At the tissue level, molecular and biochemical analyses of endogenous and transgene‐derived A‐type lamin expression were performed. As expected, quantitative PCR of endogenous and transgene‐derived transcripts demonstrated only murine lamin A/C transcripts in wild‐type (*LMNA* ^+/+^) tissues, including skin, inguinal fat, heart, and aorta (Figure S2a). In all tissues that were tested, the levels of endogenous *Lmna* transcripts were comparable across all genotypes. In both *LMNA* ^G/+^ and *LMNA* ^G/G^ tissues, however, we found variable levels of transgene expression.

Transgene expression levels correlated with copy number in skin and heart tissue. Conversely, transgene expression was significantly decreased in inguinal fat and aorta tissue of *LMNA* ^G/G^ versus *LMNA* ^G/+^ mice which may reflect loss of viable adipocytes and VSMCs, respectively, that express high levels of A‐type lamins (Uhlen et al., 2015).

Immunoblots of tissue‐derived A‐type lamins also revealed variable levels of total lamin A, progerin, and lamin C (Figure S2b). We found that, only in skin and aorta, total levels of A‐type lamins correlated with endogenous and transgene copy number. In heart and inguinal adipose tissue derived from *LMNA* ^G/G^ mice, total A‐type lamins were reduced approximately 25% and 50% in fat and heart, respectively, compared to *LMNA* ^G/+^ mice. However, total levels of endogenous and transgene‐derived A‐type lamins relative to the reference protein were consistently higher in tissues derived from transgenic mice compared to wild type (Figure S2b).

**FIGURE 3:**
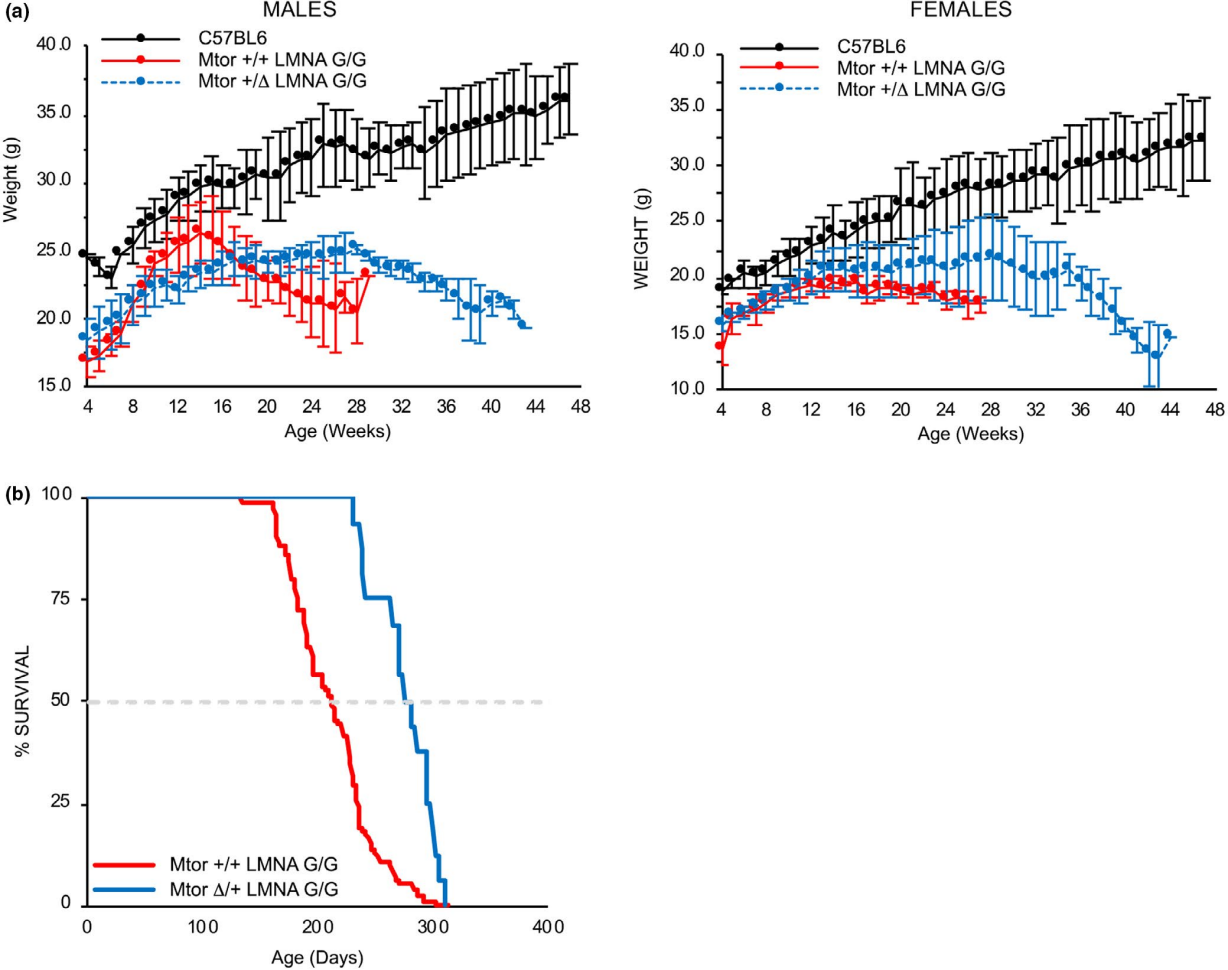
Genetic reduction of *Mtor* extends lifespan in LMNA G608G transgenic mice. (a) Growth curves of wild‐type (*Mtor* ^+/+^ *LMNA* ^+/+^), and double‐copy transgenic mice containing two wild‐type *mTor* alleles (*Mtor* ^+/+^ *LMNA* ^G/G^) or heterozygous for the *Mtor* hypomorphic allele (*Mtor* ^Δ^^/+^ *LMNA* ^G/G^). Growth deficiency in *LMNA* ^G/G^ mice is not rescued by reduced expression of mTOR in male (n = 6 *Mtor* ^+/+^ *LMNA* ^+/+^, 7 *Mtor* ^+/+^ *LMNA* ^G/G^, 13 *Mtor* ^Δ^^/+^ *LMNA* ^G/G^) or female (n = 6 *Mtor* ^+/+^ *LMNA* ^+/+^, 6 *Mtor* ^+/+^ *LMNA* ^G/G^, 12 *Mtor* ^Δ^^/+^ *LMNA* ^G/G^) mice. (b) Kaplan‐Meier plot demonstrates the 30% extension in lifespan of double‐copy transgenic mice heterozygous for the *Mtor* hypomorphic allele (*Mtor* ^Δ^^/+^ *LMNA* ^G/G^) compared to transgenic mice harboring two wild‐type *Mtor* alleles (*Mtor* ^+/+^ *LMNA* ^G/G^). *p* < 0.001

Having established by phenotypic and biochemical analyses that mice harboring two copies of the human LMNA G608G transgene represent a relevant model of classic HGPS, we turned our attention to ways in which this model might be used to identify possible therapeutic interventions to treat children with HGPS. Two treatments currently being tested include farnesyltransferase inhibitors to reduce production of the toxic progerin protein and autophagy activation with rapamycin analogs to promote its clearance (Evangelisti et al., 2016; Harhouri et al., 2018; Young et al., 2013). While the former approach has proven clinically beneficial, the latter is currently the subject of a clinical trial (Gordon et al., 2012, 2018). We reasoned that, using our mouse model, a genetic approach could be undertaken to investigate mTOR inhibition as a potential strategy to alleviate HGPS pathology. Mice carrying an *Mtor* hypomorphic allele (*Mtor* ^Δ/+^) were bred into the G608G transgenic mouse line, followed by matings of doubly heterozygous mice (*Mtor* ^Δ^^/+^ *LMNA* ^G/+^). The offspring distribution from these matings was significantly skewed toward *Mtor* ^+/+^, regardless of *LMNA* genotype, and the presence of the hypomorphic allele increased embryonic lethality from 12% to 61% in *LMNA* ^G/G^ offspring (Figure S3). As demonstrated previously, *Mtor* ^+/+^ *LMNA* ^G/G^ mice consistently weighed significantly less than their wild‐type littermates by 5 weeks (Figure 3a). Although *Mtor* ^Δ^^/+^ *LMNA* ^G/G^ mice failed to attain weights similar to wild‐type littermates, after 20 weeks of age both male and female mice harboring one copy of the *Mtor* hypomorphic allele retained 10–15% more of their weight compared to *Mtor* ^+/+^ *LMNA* ^G/G^ mice (*p *< 0.05).

Histologic examination of vascular tissue revealed quantifiable alterations in these mice (Figure 2). By five months of age, aortas of both *Mtor* ^+/+^ *LMNA* ^G/G^ and *Mtor* ^Δ^^/+^ *LMNA* ^G/G^ mice exhibited vascular alterations, including loss of vascular smooth muscle cells, deposition of proteoglycan and elastin disorganization within the medial layer, and expansion of the adventitial matrix, compared to wild‐type aortas (Figure 2a–c). However, *LMNA* ^G/G^ mice carrying the *Mtor* hypomorphic allele (*Mtor* ^Δ^^/+^ *LMNA* ^G/G^) retained twice as many VSMC within the medial layer of ascending aorta compared to *LMNA* ^G/G^ mice with normal *Mtor* expression (*Mtor* ^+/+^ *LMNA* ^G/G^.). In contrast, *Mtor* expression levels made no difference in the expansion of adventitial tissue in *LMNA* ^G/G^ mice. Importantly, the improved retention of VSMC in *Mtor* ^Δ^^/+^ *LMNA* ^G/G^ mice was associated with a 30% increase in lifespan versus *Mtor* ^+/+^ *LMNA* ^G/G^ (Figure 3b). These data suggest that genetic reduction of *Mtor* expression can partially rescue the VSMC loss that is a hallmark feature of vascular pathology in HGPS mice.

**FIGURE 4:**
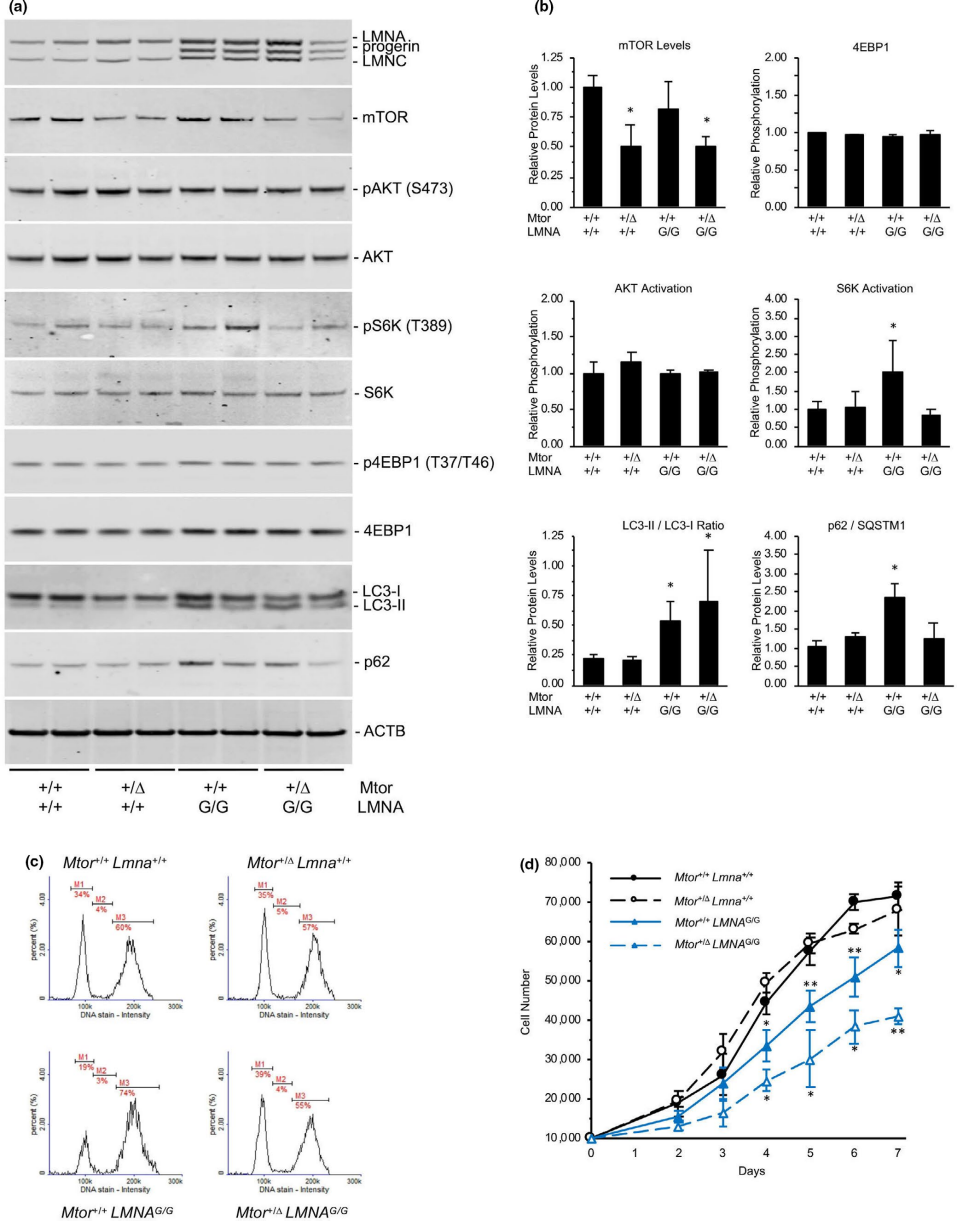
Reduction of mTOR levels in LMNA G608G newborn fibroblasts normalizes S6K activity. (a) Western analyses of A‐type lamins and mTOR signaling pathway components in fibroblast cell lines derived from *Mtor* ^+/+^ *LMNA* ^+/+^, *Mtor* ^Δ^^/+^ *LMNA* ^+/+^, *Mtor* ^+/+^ *LMNA* ^G/G^, and *Mtor* ^Δ^^/+^ *LMNA* ^G/G^ mice. (b) Quantitative analysis of mTOR signaling components from immunoblots demonstrates reduction of mTOR protein in cells heterozygous for the *Mtor* hypomorphic allele. Induction of autophagy is indicated by increased LC3‐II/I ratios in *Mtor* ^+/+^ *LMNA* ^G/G^ and *Mtor* ^Δ^^/+^ *LMNA* ^G/G^ cells versus wild‐type, and reduced levels of p62 in *Mtor* ^Δ^^/+^ *LMNA* ^G/G^ cells versus *Mtor* ^+/+^ *LMNA* ^G/G^ cells. Normalization of increased S6K phosphorylation in *LMNA* ^G/G^ cells occurs with reduced mTOR levels. *, *p *< 0.05 versus *Mtor* ^+/+^ *LMNA* ^+/+^ (c) Cell cycle analysis of proliferative fibroblasts in culture. M1, G_0_/G_1_ phase; M2, S phase; M3, G_2_/M phase. Genetic reduction of *Mtor* in transgenic cells (*Mtor* ^Δ^^/+^ *LMNA* ^G/G^) shifts the fraction of cells in G_0_/G_1_, S, and G_2_/M phases to levels seen in wild‐type (*Mtor* ^+/+^ *LMNA* ^+/+^) cells. (d) *LMNA* ^G/G^ fibroblasts are less proliferative than *LMNA* ^+/+^ cells in culture. Heterozygosity for the *Mtor* hypomorphic allele further inhibits proliferation of *LMNA* ^G/G^ murine fibroblasts. *, *p *< 0.05; **, *p *< 0.01; *Mtor* ^+/+^ *LMNA* ^G/G^ significance versus *Mtor* ^+/+^ *LMNA* ^+/+^; *Mtor* ^Δ^^/+^ *LMNA* ^G/G^ significance versus *Mtor* ^+/+^ *LMNA* ^G/G^

To determine the mechanism by which genetic reduction of mTOR elicited an increase in lifespan in *LMNA* ^G/G^ mice, fibroblast cultures were generated from dermal tissue of two independent *Mtor* ^+/+^ *Lmna* ^+/+^, *Mtor* ^Δ^^/+^ *Lmna* ^+/+^, *Mtor* ^+/+^ *LMNA* ^G/G^, and *Mtor* ^Δ^^/+^ *LMNA* ^G/G^ newborn mice for biochemical characterization of A‐type lamins and mTOR signaling pathways. As expected, no progerin was detected in cell lines expressing wild‐type *Lmna* by Western analysis (Figure 4a). In both *Mtor* ^+/+^ *LMNA* ^G/G^ and *Mtor* ^Δ^^/+^ *LMNA* ^G/G^ cell lines, the presence of progerin was associated with autophagic activation determined by the LC3‐I/II ratios. Furthermore, increased levels of autophagic cargo receptor p62/SQSTM1 in *Mtor* ^+/+^ *LMNA* ^G/G^ cultures were normalized to wild‐type levels in cells with genetically reduced *Mtor* expression suggesting autophagic elimination of this protein. However, cultures expressing the G608G transgene contained equimolar amounts of lamin A, lamin C, and progerin proteins regardless of *Mtor* allele status (Figure 4a,b). Despite the 50% reduction in intracellular mTOR protein levels and upregulation of autophagic activity in *Mtor* ^Δ^^/+^ *LMNA* ^G/G^ versus *Mtor* ^+/+^ *LMNA* ^G/G^ cell lines, no difference in the total level of A‐type lamins, or progerin as a fraction of total lamin A/C, was observed. Neither the increased basal autophagic activity nor the reduction of mTOR protein levels were associated with any reduction of nuclear blebbing, a hallmark feature of HGPS cells, in *Mtor* ^Δ^^/+^ *LMNA* ^G/G^ fibroblasts (Figure S4).

Further characterization of mTOR signaling in newborn fibroblast cultures revealed equivalent levels of phosphorylated AKT and 4EBP1 in all cell lines tested, demonstrating no difference in the extent of their activity (Figure 4a,b). In contrast, phosphorylation of S6K was increased twofold in *Mtor* ^+/+^ *LMNA* ^G/G^ compared to *Mtor* ^+/+^ *Lmna* ^+/+^, *Mtor* ^Δ^^/+^ *Lmna* ^+/+^, and *Mtor* ^Δ^^/+^ *LMNA* ^G/G^ cells (*p *< 0.05), consistent with normalization of increased S6K activity in LMNA^G/G^ fibroblasts by genetic reduction of mTOR. Normalized S6K activity in *Mtor* ^Δ^^/+^ *LMNA* ^G/G^ cells was associated with improved indices of cell growth and proliferation in comparison to *Mtor* ^+/+^ *LMNA* ^G/G^ fibroblasts; in sub‐confluent cultures, the fraction of *Mtor* ^+/+^ *LMNA* ^G/G^ cells in G_2_/M phase was increased 23%, with a corresponding 44% decrease in G_0_/G_1_ phase, when compared to *Mtor* ^+/+^ *Lmna* ^+/+^ wild‐type cells (Figure 4c). Genetic reduction of mTOR in *Mtor* ^Δ^^/+^ *LMNA* ^G/G^ cells, however, doubled the fraction of cells in G_0_/G_1_ phase while reducing the fraction of cells in G_2_/M phases by 26%, thereby altering the cell cycle distribution to more closely resemble that of *Mtor* ^+/+^ *Lmna* ^+/+^ wild‐type cells. Delayed cell growth was observed in *Mtor* ^+/+^ *LMNA* ^G/G^ fibroblasts, with an approximate doubling time of 2.5 days versus a 2‐day doubling time for wild‐type (*Mtor* ^+/+^ *Lmna* ^+/+^) cells (Figure 4d). Genetic reduction of *Mtor* had no effect on growth of wild‐type cells, but further decreased proliferation in progerin‐expressing (*Mtor* ^Δ^^/+^ *LMNA* ^G/G^) fibroblasts to a doubling time of 3.5 days.

**FIGURE 5:**
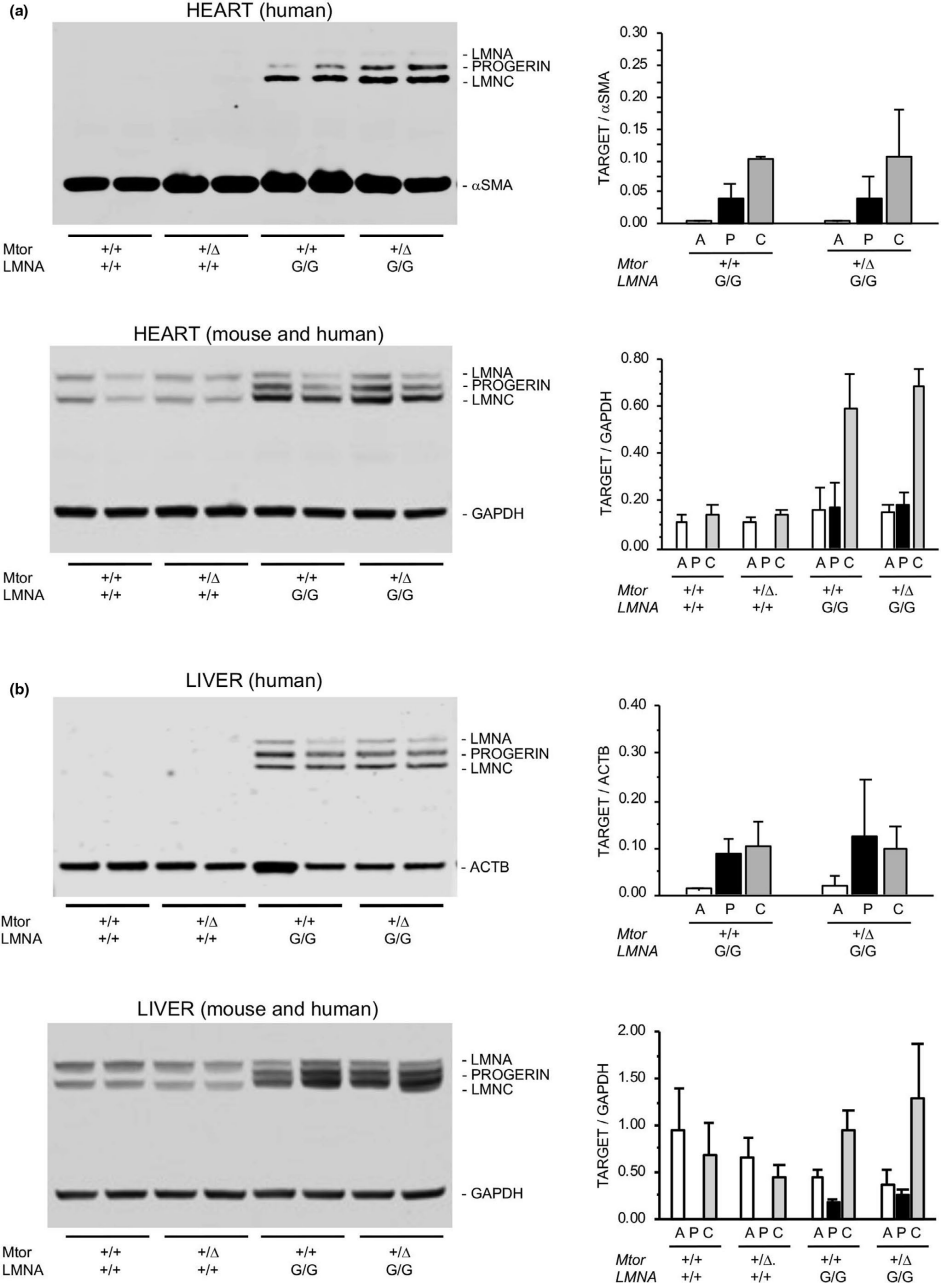
Analysis of A‐type lamins in tissues. (a, b) Western analysis of A‐type lamins extracted from heart tissue and livers of *Mtor* ^+/+^ *LMNA* ^+/+^, *Mtor* ^Δ^^/+^ *LMNA* ^+/+^, *Mtor* ^+/+^ *LMNA* ^G/G^, and *Mtor* ^Δ^^/+^ *LMNA* ^G/G^ mice (n = 2 per genotype, 1 male and 1 female). Samples in upper immunoblots were immunoprecipitated with antibodies to human lamin A/C [JoL2] and smooth muscle actin (SMA) or beta‐actin (ACTB), then probed with an alternative antibody recognizing both mouse and human Lamin A/C and reference proteins SMA or ACTB. Lower immunoblots contain tissue homogenates probed with antibody recognizing both mouse and human Lamin A/C [4c11] and GAPDH. No significant difference in lamin A, progerin, or lamin C levels was observed in mice with genetic reduction of mTOR regardless of sample preparation procedure, antibody used for detection, or reference protein used for normalization

Based on our initial hypothesis that reduced levels of *Mtor* expression would upregulate autophagy and, therefore, decrease levels of progerin *in vivo*, we analyzed tissues from *Mtor* ^+/+^ *Lmna* ^+/+^, *Mtor* ^Δ^^/+^ *Lmna* ^+/+^, *Mtor* ^+/+^ *LMNA* ^G/G^, and *Mtor* ^Δ^^/+^ *LMNA* ^G/G^ littermates. Using an antibody specifically recognizing human LMNA (JoL2), no difference in the levels of transgene‐derived lamin A, progerin, or lamin C relative to alpha‐smooth muscle actin (aSMA) or beta‐actin were found by Western analyses of immunoprecipitated A‐type lamins from heart and liver, respectively, of *Mtor* ^+/+^ *LMNA* ^G/G^ and *Mtor* ^Δ^^/+^ *LMNA* ^G/G^ mice (Figure 5a,b). Subsequent detection with an alternative antibody (4c11) reactive to both human and mouse A‐type lamins also failed to demonstrate any discernible quantitative differences in tissue homogenates derived from transgenic mice with genetically reduced *Mtor*. We therefore considered the possibility that, *in vivo*, autophagic elimination of progerin might be affected by transgene‐derived *RAB25* expression due to its putative role in regulating autophagy by activation of AKT (Cheng et al., 2012).

Quantitative RT‐PCR demonstrated that human *RAB25* expression was 2.5‐ to 60‐fold greater than endogenous mouse *Rab25*, depending on the tissue analyzed, in mice harboring two transgene copies (Figure S5a). To determine if high levels of transgene‐derived RAB25 were inhibiting autophagy, we next generated murine embryonic fibroblasts from the offspring of *Mtor* ^Δ^^/+^ mice crossed with either *Lmna* ^G609G^ knock‐in mice, which lack human *RAB25* expression, and *LMNA* ^G608G^ transgenic mice. Western analyses from cell lysates demonstrated a modest reduction in total A‐type lamins in both *LMNA* ^G608G/G608G^ transgenic (G/G) and *Lmna* ^G609G/G609G^ knock‐in (KI/KI) cell lines treated with everolimus (RAD001), as well as in cell lines harboring the *Mtor* hypomorphic allele (Figure S5b). The decrease in A‐type lamins relative to ACTB levels, which was not specific to progerin, was associated with increased LC3‐II/LC3‐I ratios and decreased p62 levels, indicating autophagic activation in cell lines where mTOR was either chemically inhibited or genetically reduced (Figure S5c).

**FIGURE 6:**
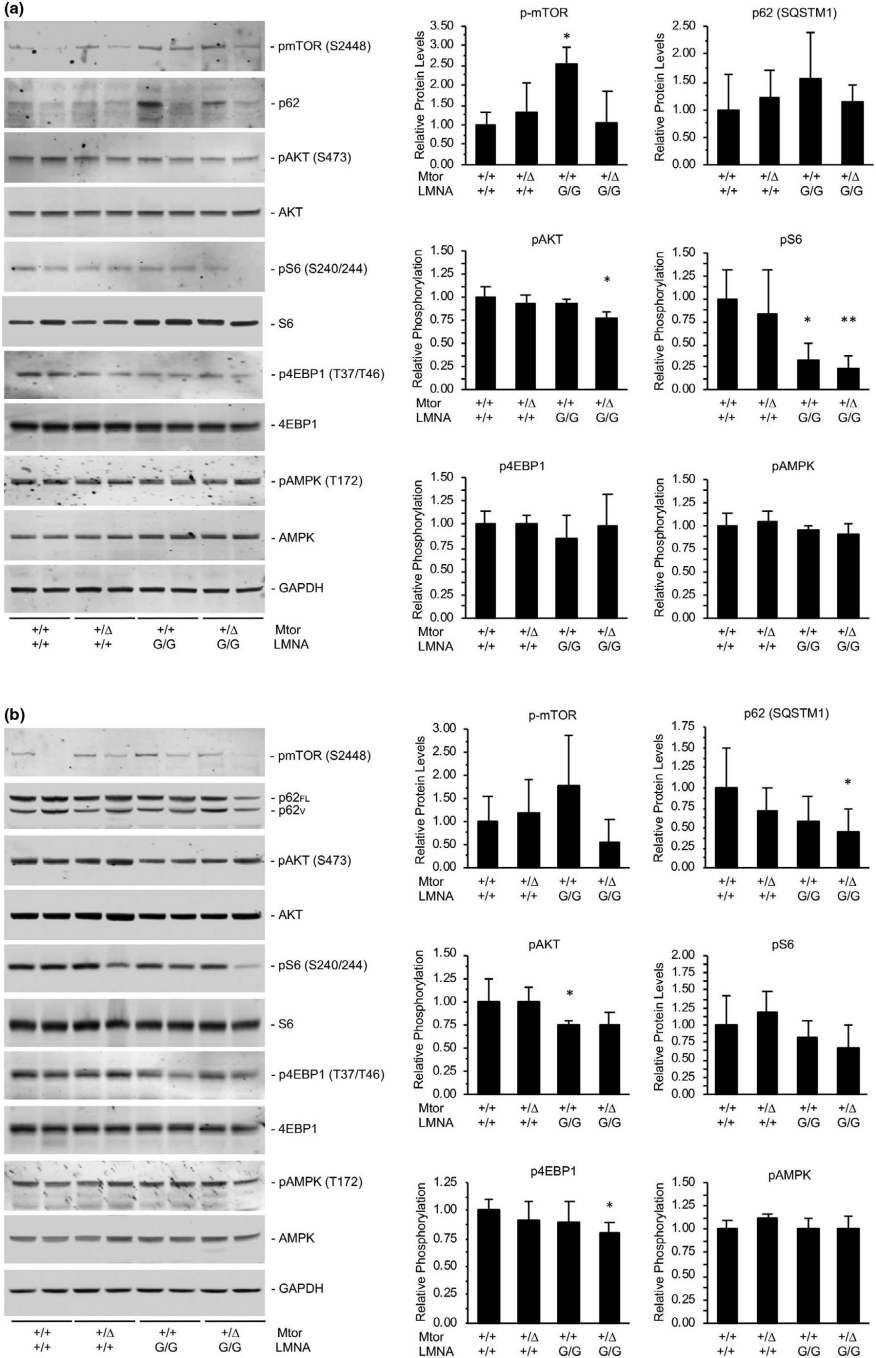
mTOR signaling pathway is inhibited in adult *LMNA* G608G mice *in vivo*. (a) Representative immunoblots of mTOR signaling pathway components isolated from heart tissue of 5 month‐old *Mtor* ^+/+^ *LMNA* ^+/+^, *Mtor* ^Δ^^/+^ *LMNA* ^+/+^, *Mtor* ^+/+^ *LMNA* ^G/G^, and *Mtor* ^Δ^^/+^ *LMNA* ^G/G^ mice. Despite hyperactivation of mTOR in *Mtor* ^+/+^ *LMNA* ^G/G^, relative phosphorylation of S6 decreased versus wild type. (b) Western analyses of liver homogenates. Genetic reduction of *Mtor* significantly reduces levels of p62 isoforms (p62_FL_, full length; p62_V_, variant (Kageyama et al., 2018)) and phosphorylated 4EBP1 in *LMNA* ^G/G^ mice. N = 4 mice per genotype (2 males, 2 females); *, *p *< 0.05; **, *p *< 0.01, *Mtor* ^+/+^ *LMNA* ^G/G^ and *Mtor* ^Δ^^/+^ *LMNA* ^G/G^ significance versus *Mtor* ^+/+^ *LMNA* ^+/+^

Analysis of mTOR signaling in tissues collected from adult mice contrasted with the results from cell cultures. Although Western immunoblots of tissue homogenates collected from 5 month‐old *LMNA* ^G/G^ mice revealed significantly increased levels of phosphorylated mTOR in hearts (Figure 6a), and to a lesser extent in livers (Figure 6b), compared to wild‐type littermates, downstream targets of mTOR signaling were found to be hypophosphorylated. In heart tissue from adult mice, relative phosphorylation of S6 was significantly reduced in *LMNA* ^G/G^ versus wild type, with further reduction in phosphorylation occurring in transgenic mice with genetically reduced *Mtor* (Figure 6a). A similar, but nonsignificant, trend in reduced S6 phosphorylation was found in livers of adult mice, where genetic reduction of *Mtor* was associated with hypophosphorylation of 4EBP1 (Figure 6b). In both tissues, we found that AKT activation was suppressed in the presence of progerin. From these data, we conclude that mTOR signaling is hyperactivated in *LMNA* ^G/G^ newborn and embryo‐derived cell cultures, but in tissues of mice where the HGPS phenotype has progressed mTOR signaling is inhibited.

## 3 DISCUSSION

Here, we describe a double‐copy *LMNA^G^* ^/^ *^G^* transgenic mouse model of HGPS that replicates most of the features of the human disorder. Several other mouse models have been created to characterize the pathogenic mechanisms underlying HGPS, and the comparison of phenotypes is useful. Yang and colleagues generated a knock‐in model, *Lmna* ^HG^, created by deletion of intron 10, intron 11 and the last 150 nucleotides of exon 11, so that only the mutant progerin transcript is produced without any normal lamin A or C (Yang et al., 2005). *Lmna* ^HG/+^ mice, which have an average lifespan of approximately 27 weeks, develop growth deficiency, bone dysplasia, and kyphosis by 4 months of age. However, somewhat surprisingly these mice do not exhibit vascular smooth muscle cell loss and the intima, media, and adventitial layers of aortas appear normal out to 7 months of age (Yang et al., 2005). In contrast, mice homozygous for the progerin‐only allele (*Lmna* ^HG/HG^) rapidly develop a more severe phenotype, characterized by spontaneous fractures and complete absence of adipose tissue with death at 3–4 weeks of age (Yang et al., 2006).

A second, well‐characterized, knock‐in model that carries the murine equivalent of the common human mutation (*Lmna* ^G609G^, c.1827C > T) produces progerin via abnormal splicing as occurs in HGPS patients. Heterozygous *Lmna* ^G609G^^/+^ mice have normal weight and size until approximately 24 weeks of age and then start to show symptoms of aging until death at an average age of 35 weeks. *Lmna* ^G609G^^/^^G609G^ mice show a more pronounced and earlier progeroid phenotype than heterozygous *Lmna* ^G609G^^/+^ mice and, prior to premature death at 14–15 weeks of age, display the main clinical manifestations of human HGPS such as osteoporosis, loss of principal fat deposits, and VSMC depletion, as well as aberrant hormonal profiles that manifest as hypoglycemia and IGF deficiency (Osorio et al., 2011).

In cell cultures from patients with HGPS, a minor fraction of transcripts derived from the mutant *LMNA* allele encode progerin, while the majority of transcripts yields mostly wild‐type lamin A and lamin C (Eriksson et al., 2003). In contrast, we found that a significant proportion of murine transcripts derived from the mutant human *LMNA* transgene encode progerin. Nevertheless, the dominant‐negative effects of progerin accumulation progress with age in both patients and murine models of HGPS. Despite the possibility that mice require a higher level of progerin expression to elicit the HGPS disease phenotype (Yang et al., 2005), it is apparent that the severity of the progeroid phenotype in *LMNA* ^G^^/^^G^ transgenic mice, as well as other mouse models of HGPS, correlates with gene dosage. Comparison to other mouse models of HGPS shows that *LMNA* ^G^^/^^G^ transgenic mice present with a less severe phenotype than *Lmna* ^G609G/G609G^ and *Lmna* ^HG/HG^ mice, which may be attributed to altered molecular interactions between human LMNA/progerin and endogenous murine nuclear proteins, or alternatively, retention of normal functioning of endogenous *Lmna* in transgenic mice.

We also noted that at the transcript level, inguinal fat and aorta from *LMNA* ^G/G^ mice expressed lower levels of progerin compared to *LMNA* ^G/+^, which may reflect the loss of specific cell populations with higher transgene expression in these tissues (Uhlen et al., 2015). While a dramatic absence of subcutaneous adipose tissue was observed, *LMNA* ^G/G^ transgenic mice do not develop alopecia or glandular and follicular abnormalities as occurs in *Lmna* ^G609G^ mice (Osorio et al., 2011).

Although *LMNA* ^G/+^ mice appear to lack any detectable bone phenotype at 6 months of age, micro‐CT analysis of *LMNA* ^G/G^ mouse femora detected a modest decrease (~5%) in BMD with significant reductions in structural parameters of trabecular and cortical bone, consistent with previous reports (Cubria et al., 2020). This aspect of the HGPS phenotype appears to be due to defective modeling of bone tissue, since bone‐ and cartilage‐specific *Osx*‐driven expression of progerin leads to skeletal abnormalities associated with impaired osteoblast differentiation and loss of the osteocyte population in bone tissue (Schmidt et al., 2012). While there is no current evidence of increased resorption, the potential for reduced cortical and trabecular bone volumes in *LMNA* ^G/G^ femurs resulting from abnormal bone remodeling cannot be ruled out. Despite key differences existing between rodent and human skeletal systems, the *LMNA* ^G/G^ transgenic mouse model will be a useful model for further investigating the effects that progerin expression has on bone cell populations in HGPS bone dysplasia and, potentially, its role in aging‐related osteoporosis.

As was seen in *LMNA* ^G/+^ mice, *LMNA* ^G/G^ mice were found to have a progressive and dramatic defect of large arteries, particularly the aorta. These pathologic alterations in the medial layer of large vessels of transgenic mice recapitulate findings in HGPS. Since SM2a‐Cre‐mediated VSMC‐specific expression of progerin is sufficient to accelerate pathologic vascular alterations and death in atheroprone Apolipoprotein E‐null mice (Hamczyk et al., 2018), progerin‐induced vascular pathology may be directly related to the cause of death in patients. Although the precise mechanism(s) underlying the vascular changes remains to be clarified, HGPS VSMCs and endothelial cells are more sensitive to mechanical stress, have been shown to be particularly susceptible to progerin accumulation and are exposed to the higher hemodynamic forces (pressure and shear stress) exerted on arterial walls (Varga et al., 2006; Verstraeten et al., 2008), which may be influenced by dysregulation of the glycocalyx components involved in flow shear stress sensing (Pitrez et al., 2020). Progerin‐induced VSMC vulnerability has also been attributed to dysregulation of the DNA damage response pathway, hyperactivation of ER stress (ER), and the unfolded protein response (UPR) (Hamczyk et al., 2019; Kinoshita et al., 2017; Zhang et al., 2014). Furthermore, it can be speculated that the adventitial expansion seen in *LMNA* ^G/G^ aortas is a fibrotic event mediated by VSMC‐secreted inflammatory cytokines as part of an apoptotic program or, alternatively, driven by a senescence‐associated secretory phenotype. Interestingly, disruption of the linker of the nucleoskeleton and cytoskeleton (LINC) complex in smooth muscle cells has been shown to reduce VSMC loss and adventitial fibrosis, suggesting that disruption of transmission of biomechanical forces to the nucleus may underlie these processes (Kim et al., 2018).

The alterations in tissues of mesenchymal origin, particularly skin, fat, bone and vascular tissues that we have characterized, make the *LMNA* ^G/G^ transgenic mouse a useful model for investigating therapeutic approaches to treat HGPS. Our primary goal in generating this mouse model was to enable testing of RNA‐ and DNA‐based treatments targeting the classic HGPS mutation at the gene and transcript levels in the context of the human sequence. To date, however, clinical trials have been limited to small molecule applications. For example, we have previously demonstrated that the FTI tipifarnib not only prevents onset of the cardiovascular phenotype when treatment begins at weaning, it also may be capable of inducing disease regression in LMNA^G/+^ mice that already manifest severe arteriosclerotic changes (Capell et al., 2008). In human patients, results from a trial of the farnesyltransferase inhibitor lonafarnib demonstrated benefit to the vascular system with reduction in peripheral vascular resistance and a modest improvement in survival (Gordon et al., 2012, 2018). A combination trial of lonafarnib, zoledronic acid, and pravastatin failed to show benefit beyond that obtained by lonafarnib alone (Gordon et al., 2016).

The most recent clinical trial for HGPS seeks to test the possible benefit of everolimus, an mTOR inhibitor. The rationale for this is based upon *in vitro* experiments, where administration of rapamycin substantially reversed nuclear blebbing and premature senescence in HGPS patient‐derived cell lines (Cao et al., 2011). The rapamycin analogue, everolimus, also has a demonstrated beneficial effect on the phenotype of cell lines derived from patients with various laminopathies caused by mutations in *LMNA* (DuBose et al., 2018). These mechanistic studies have documented that the beneficial effects appeared to be based not only upon enhanced autophagic clearance of progerin aggregates, but also normalization of the levels of phosphorylated RPS6, a downstream target of S6 kinase (Cao et al., 2011; DuBose et al., 2018). Considering this background, we have investigated inhibition of mTOR signaling as a potential treatment for HGPS.

In a genetic strategy to test the effect of mTOR inhibition in our mouse model, mice carrying an *Mtor* hypomorphic allele (*Mtor* ^Δ/+^) were bred into the G608G transgenic mouse line. We found no significant improvement of nuclear membrane structure in newborn fibroblasts derived from offspring of these matings, nor could we verify any reduction of progerin in tissues of *Mtor* ^+/+^LMNA^G/G^ mice in the presence of activated autophagic activity in adult tissues. Importantly, however, genetic reduction of *Mtor* in HGPS transgenic mice led to a 30% increase in lifespan versus *Mtor* ^+/+^ *LMNA* ^G/G^ littermates.

Analysis of mTOR signaling in fibroblasts derived from newborn mice demonstrated that increased activation of the downstream effector S6 protein kinase (S6K) in *Mtor* ^+/+^ *LMNA* ^G/G^ was normalized in *Mtor* ^Δ/+^ *LMNA* ^G/G^, consistent with lifespan extension observed in S6K‐null mice (Selman et al., 2009). Genetic reduction of *Mtor* has previously been reported to normalize increased mTOR/S6K1 activity, partially restore autophagy and postpone the premature aging phenotype in *Zmpste24*‐null mice (Pan et al., 2020). Hyperactivation of S6K has also been observed in adipocyte precursor and osteosarcoma cell lines expressing *LMNA* mutations associated with Type 2 Familial Partial Lipodystrophy (FPLD2) and Mandibuloacral Dysplasia (MADA), respectively (Evangelisti et al., 2015; Pellegrini et al., 2019). Moreover, there is evidence to suggest that S6K mediates the pathogenesis and premature death of *Lmna*‐null mice, and reducing its activity improves muscle function as well as lifespan (Liao et al., 2017).

In contrast to cells from embryos and newborn mice, however, signaling downstream of mTOR was found to be inhibited in *LMNA* ^G/G^ adult tissues, particularly in the heart. These findings suggest that hyperactivation of mTOR signaling may be an early event in the onset of HGPS pathology in *Mtor* ^+/+^ *LMNA* ^G/G^ mice, but its activity is age‐, phenotype‐, and tissue‐dependent, similar to what has been observed for *Zmpste24* ^−/−^ mice (Pan et al., 2020), and which may be informative to the rational design of a therapeutic approach involving chemical inhibition of mTOR signaling.

The mechanism by which reduction of S6K activity increases lifespan in our mouse model of HGPS remains to be determined. Interaction of S6K1 with various substrates promotes several broad cellular processes such as protein production, cell growth and size, cell survival, and gene transcription. For example, S6K1 regulates protein production by phosphorylating substrates that function in translational initiation, elongation, and protein folding, including eIF4B, PDCD4, SKAR, CBP80, eEF2K, CCTβ, and RPS6, a component of the 40S ribosome. S6K1 also regulates transcription by activating CREM, estrogen receptor, and SREBP (Duvel et al., 2010; De Groot et al., 1994; Yamnik et al., 2009). Furthermore, S6K1 participates in the DNA damage response by phosphorylating MDM2, thereby blocking its nuclear import and ability to ubiquitinate the tumor suppressor p53 (Lai et al., 2010). Finally, S6K1 promotes negative feedback on PI3K signaling through IRS1, thus suppressing insulin and IGF sensitivity (Tremblay et al., 2007). It will be important, therefore, to determine which specific signaling events downstream of S6K1 are altered in response to genetic reduction of *Mtor* in our mouse model of HGPS.

These data provide further evidence, based on an animal model carrying the human progeria mutation, that inhibitors of mTOR might provide benefit for children with HGPS. It should be kept in mind, however, that the mTOR protein takes part in two different complexes, mTORC1 and mTORC2. Only the first of these is classically stated to be sensitive to rapamycin and its homologs. So the genetic approach we describe here, which reduces function of both mTORC1 and mTORC2, is not a perfect analog of the pharmacologic blockage of mTORC1 that is being pursued with an everolimus trial.

In summary, we have generated a murine model that is well suited for testing therapeutic interventions to treat Hutchinson‐Gilford progeria syndrome in the context of the human gene and protein. Furthermore, this study supports a strategy for pharmacological inhibition of a specific component of the mTOR signaling pathway as a potential treatment of HGPS.

## 4 EXPERIMENTAL PROCEDURES

### 4.1 Mouse strains and animal care

A transgenic mouse model of HGPS was developed by retrofitting a human bacterial artificial chromosome (BAC) harboring the *LMNA* gene containing the classic G608G mutation (c.1824C > T). The 164 kb transgene was incorporated into the germline of C57BL/6J mice as previously described (Varga et al., 2006). Single‐copy mice, designated *LMNA* ^G/+^, were bred within the C57BL/6J line for twenty generations prior to generation of double‐copy mice, designated as *LMNA* ^G/G^, for experimental use. Mice harboring the *Mtor* hypomorphic allele (*Mtor* ^Δ^^/+^) were a generous gift from Beverly Mock, National Cancer Institute, and have been previously characterized (Wu et al., 2013). Mice harboring the *Lmna* ^G609G^ knock‐in mutation were a generous gift from Carlos López‐Otín, Universidad de Oviedo (Osorio et al., 2011). Murine growth curves were determined by weekly weights. Kaplan‐Meyer plots were generated from in‐house data combined with published wild‐type C57BL/6J data from Jackson Laboratories (https://www.jax.org/jax‐mice‐and‐services/strain‐data‐sheet‐pages/body‐weight‐chart‐000664). Animal care and experiments were performed in accordance with a protocol approved by the NHGRI Animal Care and Use Committee.

### 4.2 Cell cultures

Primary fibroblast cultures (FB) were derived from dermal tissue dissected from the abdomens of newborn mice. FB were allowed to grow out from dermal samples for 2 weeks before releasing by trypsin digestion. Murine embryonic fibroblasts (MEFs) were isolated from day 14–15 (E14‐E15) embryos generated by crossing G608G transgenic or G609G knock‐in mice with mice expressing the *Mtor* hypomorphic allele. After skin and viscera were removed, embryos were minced and digested in 0.25% trypsin for 15 min. Cells were plated and allowed to adhere for 24 h at 37℃. The following day, non‐adherent cells were removed, and the adherent cell population was expanded for collection as MEFs. Cells were cultured in DMEM containing 10% fetal bovine serum, 2 mM glutamine, and 1% pen‐strep at 37℃ in 5% CO_2_. Cells cultured with everolimus (RAD001) were treated at 0.1 μM for 4 h prior to collection.

### 4.3 Proliferation assays

Cells were plated at 10,000 cells per well in 24‐well culture plates (day 0) in triplicate. Culture medium was replaced daily during the course of the assay. Each cell line was collected by trypsinization at 24‐h increments. Cell concentrations were determined in Via‐1 cassettes using a Nucleocounter with Nucleoview‐3000 software (Chemometec). Cell number was calculated and plotted using Microsoft Excel software.

### 4.4 Western blot analyses

Cell cultures and tissues were homogenized in high‐salt RIPA buffer (20 mM Tris‐HCl, pH7.4; 0.5 M NaCl; 1mM EDTA; 0.1% SDS, 1% Triton X‐100, 1x protease inhibitor (Sigma, P8340), 100 mM AEBSF (Sigma, SBR00015), 20 μM caspase inhibitor VI (Sigma, 219007)) and quantitated by BCA assay. Initial loads used 20 μg of total protein before balancing according to reference proteins. Lysates were electrophoresed on 3–8% Tris‐acetate (mTOR), 4–12% Bis‐Tris (LC3A/B), or 8% Bis‐Tris gels (Thermo Fisher), transferred to nitrocellulose membranes, which were blocked in 5% milk or 5% BSA overnight at 4℃. Blots were incubated with primary antibody for 4 h at room temperature, followed by 2 h incubation with IRDye® 800CW donkey anti‐mouse IgG or IRDye® 680RD donkey anti‐rabbit IgG fluorescent secondary antibodies (LI‐COR Biosciences) and imaged on an Odyssey CLx Imaging system with Image Studio software (LI‐COR Biosciences). A list of all antibodies used in this study is available in Supplementary Methods.

### 4.5 Cell cycle analysis

Subconfluent fibroblast cultures were collected by trypsinization and pelleted at 500 × *g* for 10 min. Cells were then washed once with PBS and centrifuged again at 500 × *g* for 10 min. Cell pellets were resuspended in PBS and fixed in a final concentration of 70% EtOH overnight at 4℃. The following day cells were centrifuged at 500 × *g* for 5 min, resuspended in PBS for 1 min to rehydrate, then centrifuged again for an additional 5 min. Cells were gently resuspended in propidium iodide staining solution (20 μg/ml PI, 0.1% triton X‐100, 200 μg/ml RNase A in PBS) and incubated at 37℃ for 30 min. Stained cells were analyzed using the Fixed Cell Cycle‐PI Assay on a NucleoCounter NC‐3000 (Chemometec).

### 4.6 Histology

Tissues were fixed in 2% paraformaldehyde for 24 h prior to dehydration with graded alcohols and embedding in paraffin. Cross‐sections (4‐μm thick) were cut and mounted on charged slides and visualized by Masson's trichrome, hematoxylin/eosin, Movat's pentachrome, or Picrosirius red staining (CV Path Inc). Images were captured on a Axioscan imaging system (Zeiss) at 20× magnification and were further processed by trimming using ZenBlue microscopy (Zeiss) and Photoshop CC (Adobe) software.

### 4.7 Bone structural analysis

Left femora of 6‐month‐old wild‐type (*LMNA* ^+/+^, 10 males, 9 females), single‐ (*LMNA* ^G/+^, 10 males, 9 females), and double‐copy transgenic *(LMNA* ^G/G^, 10 males, 10 females) littermates were analyzed by micro‐computed tomography (μCT40, Scanco Medical AG). Sequential transaxial images were generated using an integration time of 250ms and a tube voltage and current of 70kVp and 114mA, respectively. Images were reconstructed at 10 μm isotropic voxel size, then assessed for volumetric bone mineral density (vBMD, mg/cm^3^) using calibrated hydroxyapatite (HA) phantoms to convert X‐ray attenuation to a known mineral density. Morphometric analyses were performed using native Scanco software. The trabecular region of interest (ROI) was located proximal to the distal femoral growth plate and extended 150 slices upward toward the proximal end. The diaphyseal cortical ROI started on the 55 percentile femur height from the proximal end, spanning 50 slices toward the distal end.

## CONFLICT OF INTEREST

The authors declare no competing interests.

## AUTHOR CONTRIBUTIONS

WAC, MRE, AN, and FSC designed the experiments, which were conducted by WAC, IB, DY, UT, and YDB. Data were analyzed by WAC, IB, DY, UT, YDB, MAE, AN, and MRE. Manuscript was written by WAC, MRE, and FSC with significant input from MAE and AN.

## Supporting information

Supplementary Material

## Data Availability

The data that support the findings of this study are available from the authors upon request.
