# Supplementary material for: Genetic reduction of mTOR extends lifespan in a mouse model of Hutchinson‐Gilford Progeria syndrome
Source: Aging Cell. 2021 Aug 28;20(9):e13457. doi: 10.1111/acel.13457 (PMC8441492; doi:10.1111/acel.13457)
Supplement: Supplementary file 1 — Supplementary Material [file ACEL-20-e13457-s001.docx]

**Supporting Information**

**Supplementary Methods**

**Transgenic BAC locus structure determination.** High molecular weight genomic DNA was extracted from 1x10^6^ low passage mouse embryonic fibroblast cells (MEFs) from *LMNA*^G/G^ mice. Long read whole genome DNA sequencing (WGS) was performed using PromethION technology (Oxford Nanopore Technologies). Sequence reads containing the human BAC transgenic sequence of RP11-702H12 were extracted from *LMNA*^G/G^ WGS output using MashMap (Jain, Koren, Dilthey, Phillippy, & Aluru, 2018) and the subset was assembled using Canu (Koren et al., 2017) and Miniasm (Li, 2016) for reproducibility. The Canu generated sequence ends were localized to the mouse genome sequence GRCm38.4 using BLAST (NCBI). Homologous sequence alignment between the *LMNA*^G/G^ human BAC transgenic contiguous assembly and the original RP11-702H12 human BAC was performed using MUMmer (Delcher, Phillippy, Carlton, & Salzberg, 2002).

**Gene Expression Analyses.** Murine tissues were collected into Trizol reagent (ThermoFisher), homogenized and immediately flash-frozen until ready for total RNA isolation. RNA was subsequently digested for 20 minutes at 37^o^C with recombinant DNase I (ThermoFisher), then analyzed for integrity and concentration on an Agilent nucleic acid bioanalyzer (Agilent Technologies). Synthesis of cDNA utilized the iScript cDNA Synthesis kit (BioRad) according to the manufacturer's protocol. For each transcript assay, droplets were generated using 50ng of cDNA, 900nM primers, 250nM probes in 1x ddPCR^TM^ Supermix for Probes (BioRad) on a QX200 Droplet Generator, followed by PCR amplification. The sequence of primers and probes for mouse and human A-type lamins are listed in **Supplemental Table S1**. Quantitation of mouse and human *Rab25*/*RAB25* transcripts was performed with mouse Rab25 GEX Assay (assay ID #dMmuCPE5122786, FAM, BioRad) and human RAB25 GEX Assay (assay ID # dHsaCPE5036530, FAM, BioRad). PCR cycling conditions consisted of an initial enzyme activation step for 10 minutes at 95^o^C, followed by 40 cycles of 94^o^C for 30 seconds and 59^o^C for 30 seconds with a 2^o^C/second ramp rate, and a 10 minute deactivation step at 98^o^C for 10 minutes. Each reaction was duplexed with the Mouse Hprt PrimePCR^TM^ Probe Assay (assay ID #qMmuCEP0054164, HEX, BioRad), performed in triplicate. Samples were read on a QX200 droplet reader (BioRad) to obtain expression levels relative to murine *Hprt* and transcript-specific copy number, then further analyzed using Excel software. For A-type lamins, testing of assay specificity demonstrated that each human and mouse assay was species-specific using total RNA isolated from cultured classic HGPS patient and *Lmna*^G609G/+^ mouse (Osorio et al., 2011) primary fibroblasts (**Supplemental Fig S6**).

**Western Immunoblot analyses.** For analysis of tissue extracts mouse necropsies were performed at 5 months of age. Following homogenization, target proteins were pulled down using mouse anti-human Lamin A/C [JoL2, 1:50], mouse anti-β-actin [15G5A11/E2, 1:200] (ThermoFisher, MA1140) or mouse anti-α-smooth muscle actin [1A4, 1:200] (Cell Signaling Technologies, 48938) antibodies and protein G sepharose (CST, 37478). Alternatively, clarified tissue homogenates were directly loaded and electrophoresed on 4-12% Bis-Tris gels prior to transfer. For relative determination of relative phosphorylation, immunoblots were loaded for a balanced signal for total protein, stripped and reprobed for phosphor-specific antibody.

The primary antibodies and concentrations used for immunoblots in this study were as follows: mouse anti-human Lamin A/C [JoL2] (1:50, Abcam, ab40567), rabbit anti-Lamin A+C [EPR4068] (1:500, Abcam, ab108922), mouse anti-Lamin A/C [4C11] (1:500, CST, 4777), rabbit anti-alpha smooth muscle actin (1:500, Abcam, ab5694), rabbit anti-β-Actin [D6A8] (1:500, CST, 8457), mouse anti-GAPDH [6C5] (1:1000, Sigma, MAB374), rabbit anti-mTOR [7C10] (1:500, CST, 2983), rabbit anti-phosphoS2448 mTOR [D9C2] (1:500, CST, 5536), rabbit anti-LC3A/B [D3U4C] (1:250, CST, 12741), rabbit anti-panAKT [C67E7] (1:500, CST, 4691), rabbit anti-phosphoS473 panAKT [D9E] (1:500, CST, 4060), rabbit anti-S6K1 [49D7] (1:500, CST, 2708), rabbit anti-phosphoT389 S6K1 [108D2] (1:500, CST, 9205), rabbit anti-p62/SQSTM1 [D1Q5S] (1:500, CST, 39749), rabbit anti-4EBP1 [53H11] (1:500, CST, 9644), rabbit anti-phosphoT37/T46 4EBP1 [236B4] (1:500, CST, 2855), rabbit anti-AMPK (1:500, CST, 2532), rabbit anti-phosphoT172 AMPK (1:500, CST, 2531), mouse anti-S6 [54D2] (1:500, CST, 2317), and rabbit anti-phosphoS235/236 S6 [D57.2.2E] (1:500, CST, 4858).

**Nuclear Blebbing Analysis.** Primary mouse fibroblasts were seeded onto 8 chamber treated culture slides (Corning, 354108) at 25,000 cells per well and allowed to attach overnight. The next day, cultures were fixed with 4% paraformaldehyde (Electron Microscopy Science, 15710) in 1X PBS and permeabilized with 0.5% Triton X-100 in 1X PBS. Slides were blocked overnight with 4% BSA in 1X TBS at 4^o^C, then incubated with primary antibody to lamin A/C (mouse anti-Lamin A/C [4c11], CST, 4777) diluted 1:200 in 4% BSA in 1X TBS overnight at 4^o^C. After repeated washes in 1X TBS, slides were incubated with secondary antibody (AlexaFluor 488 Donkey Anti-Mouse IgG, ThermoFisher, A21202 ) diluted 1:1000 in 4% BSA in 1X TBS and for 2 hours, then washed. Slides were mounted with Vectashield Mounting Media containing DAPI (Vector Laboratories, Inc., H1200) prior to imaging. Wide-field images were collected with a DeltaVision Elite system (GE Healthcare) mounted on an inverted Olympus IX71 microscope with an U Apo 20x/0.75 objective lens. Images were deconvolved in GE Healthcare’s SoftWoRx software package version 7.0, then analyzed by 5 blinded observers to quantify the presence of nuclear blebbing, with combined results used for statistical analyses.

**Kyphosis Measurements.** Whole body scans were performed at the indicated ages using an Inveon 5100 PET/CT scanner (Siemens), using an integration time of 325ms at 80kV and 500μA, respectively. Three-dimensional reconstructed images provided a resolution of 48μm. The angle of spinal curvature was determined on lateral images obtained by micro-computed tomography. Angles were calculated by intersecting spinal alignments proximal and distal to the curvature at the junction of thoracic and lumbar vertebrae (T13).

**Supplementary References**

Delcher, A. L., Phillippy, A., Carlton, J., & Salzberg, S. L. (2002). Fast algorithms for large-scale genome alignment and comparison. *Nucleic Acids Res, 30*(11), 2478-2483. doi:10.1093/nar/30.11.2478

Jain, C., Koren, S., Dilthey, A., Phillippy, A. M., & Aluru, S. (2018). A fast adaptive algorithm for computing whole-genome homology maps. *Bioinformatics, 34*(17), i748-i756. doi:10.1093/bioinformatics/bty597

Koren, S., Walenz, B. P., Berlin, K., Miller, J. R., Bergman, N. H., & Phillippy, A. M. (2017). Canu: scalable and accurate long-read assembly via adaptive k-mer weighting and repeat separation. *Genome Res, 27*(5), 722-736. doi:10.1101/gr.215087.116

Li, H. (2016). Minimap and miniasm: fast mapping and de novo assembly for noisy long sequences. *Bioinformatics, 32*(14), 2103-2110. doi:10.1093/bioinformatics/btw152

Osorio, F. G., Navarro, C. L., Cadinanos, J., Lopez-Mejia, I. C., Quiros, P. M., Bartoli, C., . . . Lopez-Otin, C. (2011). Splicing-directed therapy in a new mouse model of human accelerated aging. *Sci Transl Med, 3*(106), 106ra107. doi:10.1126/scitranslmed.3002847

**Supplementary Table 1. Primers and probes used in ddPCR assays.**

|  |  |  |
| --- | --- | --- |
| Species / Transcript | Oligo Name | Primer / Probe Sequence |
|  |  |  |
|  |  |  |
| human LMNA | hLMNA-F | CCCAGGTGGGCGGAC |
|  | hLMNA-R | AGGAGCGGGTGACCAGATT |
|  | hLMNA-FAM | 56-FAM-CAGCTACCGCAGTGTGGGGG-IABkFQ |
|  |  |  |
| human Progerin | hPROG-F | CTGTGCGGGACCTGCG |
|  | hPROG-R | AAGCCTCCACCCCCACC |
|  | hPROG-FAM | 56-FAM-AGGAGCCCAAGCCCCCAGAACT-IABkFQ |
|  |  |  |
| human LMNC | hLMNC-F | GTGGAAGGCACAGAACACCT |
|  | hLMNC-R | CATTCTTTAATGAAAAGATTTTTGG |
|  | hLMNC-FAM | 56-FAM-CAGTGACTGTGGTTGAGGACGACG-IABkFQ |
|  |  |  |
|  |  |  |
| mouse Lmna | mLmna-F | GATCCATCTCCTCTGGCTCT |
|  | mLmna-R | TGACTAGGTTGTCCCCGAA |
|  | mLmna-FAM | 56-FAM-AAGCTTCCGCAGTGTGGGGG-IABkFQ |
|  |  |  |
| mouse Progerin | mProg-F | ACAATGAGGATGACGACGAG |
|  | mProg-R | GCAGGTCCCAGATTACATGA |
|  | mProg-FAM | 56-FAM-CGGGAGCCCAGAGCTCCCAGAAC-IABkFQ |
|  |  |  |
| mouse Lmnc | mLmnc-F | TGACCATGGTTGAGGACAAT |
|  | mLmnc-R | AAAAGACTTTGGCATGGAGG |
|  | mLmnc-FAM | 56-FAM-CTGAGGCCCAGCCCACAAGG-IABkFQ |
|  |  |  |

**Supplementary Figure Legends**

**Supplementary Figure 1 Phenotypic features of LMNA G608G transgenic mice. (**a**)** Configuration of the *LMNA* G608G human transgene plotted as a one-to-one correlation of sequence between the human BAC, on the right Y-axis, and the genomic sequence of chromosome 4 in the mouse, on the x-axis, where the BAC was inserted. (b) Genotypes of offspring generated by matings between single-copy transgenic (*LMNA*^G/+^) mice were determined at weaning (3 weeks of age). The distribution suggests a minor rate (12%) of embryonic or perinatal lethality in double-copy transgenic (*LMNA*^G/G^) relative to wild-type offspring (*LMNA*^+/+^) when compared to the theoretical Mendelian distribution (p<0.0001). No significant difference was seen in generation of single-copy transgenic (*LMNA*^G/+^) mice from these matings. (c) Posterior views of 5 month-old wild-type (*LMNA*^+/+^), single-copy (*LMNA*^G/+^) and double-copy (*LMNA*^G/G^) littermates. Mice with two copies of the LMNA G608G transgene (*LMNA*^G/G^) exhibit growth deficiency relative to single-copy (*LMNA*^G/+^) and wild-type (*LMNA*^+/+^) littermates. (d) Kyphosis develops in *LMNA*^G/G^ mice by 4 months and progresses with age. * p < 0.05, ** p < 0.01. (e) Picrosirius red-stained sections of ascending aortas from 5 month-old mice show the expanded adventitial matrix comprised of collagenous matrix in *LMNA*^G/G^ versus *LMNA*^+/+^.

**Supplementary Figure 2 Expression of endogenous and transgene-derived A-type lamins in G608G transgenic mice.** (a) Quantitative PCR analysis of transcripts extracted from 5 month-old wild-type (*LMNA*^+/+^), single-copy (*LMNA*^G/+^) and double-copy (*LMNA*^G/G^) transgenic mouse tissues. Expression of the endogenous *Lmna* gene (Murine) and human *LMNA* transgene (Human) were quantitated relative to murine *Hprt* transcripts. A, lamin A; P, progerin; C, lamin C. (b) Western analyses of A-type lamins immunoprecipitated from homogenized 5 month-old wild-type (*LMNA*^+/+^), single-copy (*LMNA*^G/+^) and double-copy (*LMNA*^G/G^) transgenic mouse tissue extracts. Sample loading was balanced for beta-Actin (ACTB) or alpha smooth muscle actin (aSMA).

**Supplementary Figure 3 Offspring distribution generated by matings between double heterozygous (*Mtor*^+/Δ^*LMNA*^G/+^) mice.** No mice homozygous for the *Mtor* hypomorphic allele (*Mtor*^Δ/Δ^) were identified at weaning (3 weeks). N = 450 mice/gender; p < 0.0001.

**Supplementary Figure 4 Genetic reduction of mTOR does not reverse nuclear blebbing in LMNA G608G transgenic fibroblasts.** Confocal microscopy of wild-type (*Lmna*^+/+^) and double-copy transgenic (*LMNA*^G/G^) fibroblasts harboring two wild-type *Mtor* alleles (*Mtor*^+/+^) or heterozygous for the *Mtor* hypomorphic allele (*Mtor*^+/Δ^). Cells were stained with anti-lamin A/C antibody and visualized at 20x magnification. There were significantly more cells with blebbed nuclei in *Mtor*^+/+^*LMNA*^G/G^ cells versus *Mtor*^+/+^*Lmna*^+/+^ cells (p < 0.05). Reduction of mTOR in transgenic cells (*Mtor*^+/Δ^*LMNA*^G/G^) did not result in significant reduction of nuclear blebbing when compared to *Mtor*^+/+^*LMNA*^G/G^ cells. NS, not significant.

**Supplementary Figure 5 Transgene-derived *RAB25* overexpression does not inhibit autophagy in cell culture.** (a) Quantitative RT-PCR analysis of tissue-derived total RNA demonstrates a 2-60 fold higher level of expression of transgene-derived transcripts (hRAB25) compared to endogenous transcripts (mRab25). (b) Western analysis of cell lysates from murine embryonic fibroblasts (MEFs) derived from embryos generated by wild-type (*LMNA*^+/+^), G608G transgenic (*LMNA*^G/G^) and *Lmna*^G609G^ knock-in (KI/KI) mice crossed with Mtor hypomorphic (*Mtor*^+/Δ^) mice. Cell cultures were treated in the absence (-) or presence (+) of everolimus (RAD001) prior to collection. There is a modest reduction, relative to actin levels, of all A-type lamins in HGPS cell lines with genetically reduced or chemically inhibited mTOR. (c) Immunoblots confirm decreased levels of p62 and increased LC3-II/LC3-I ratios in HGPS cell lines with genetically reduced or chemically inhibited mTOR, consistent with autophagic activation.

**Supplementary Figure 6 Digital droplet PCR analysis of murine endogenous and human transgene-derived A-type lamins.** The specificity of each mouse and human ddPCR assay was verified using total RNA isolated from cultured classic HGPS patient and *Lmna*^G609G^ mouse fibroblasts. (a) Human-specific *LMNA* (hLMNA), progerin (hPROG) and *LMNC* (hLMNC) assays detected transcripts in RNA isolated from HGPS patient fibroblasts, but not murine fibroblasts. (b) Only murine endogenous *Lmna* transcripts were detected by ddPCR analysis of HGPS patient and *Lmna*^G609G/+^ transcripts using the mouse-specific *Lmna* (mLMNA), progerin (mPROG) and *Lmnc* (mLMNC) assays.

**Supplementary Figure 1**

**
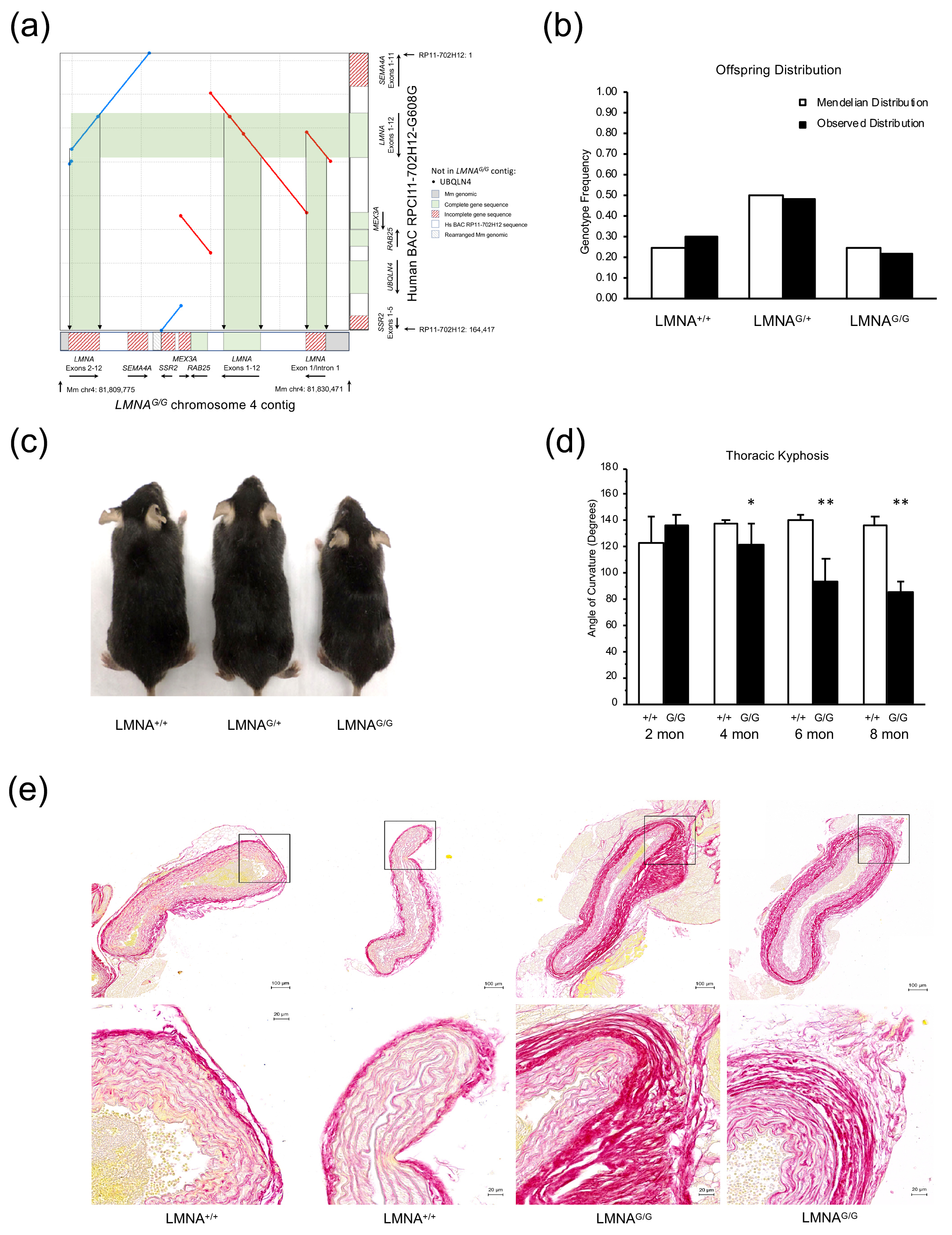
**

**Supplementary Figure 2**

**
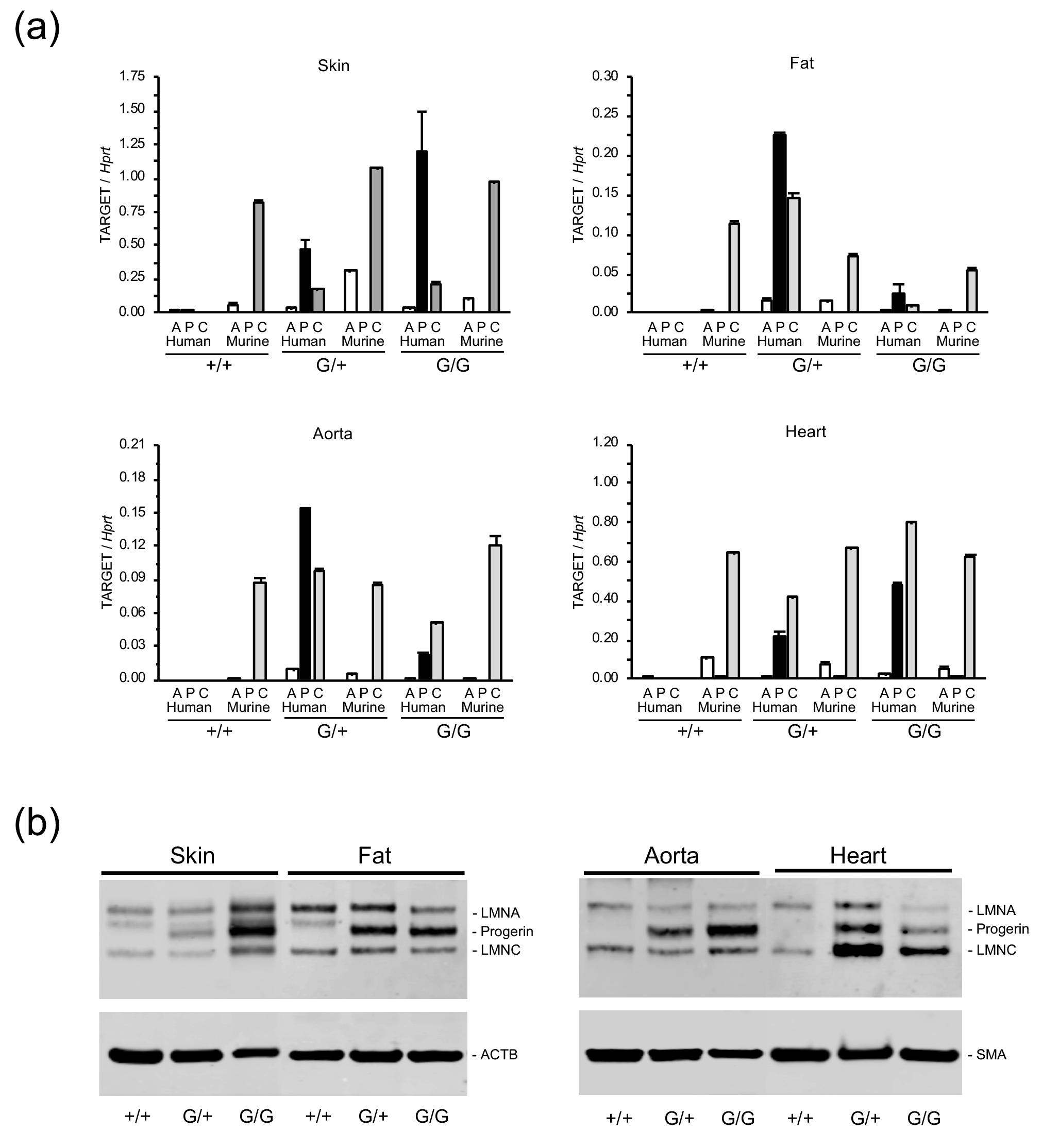
**

**Supplementary Figure 3**

**
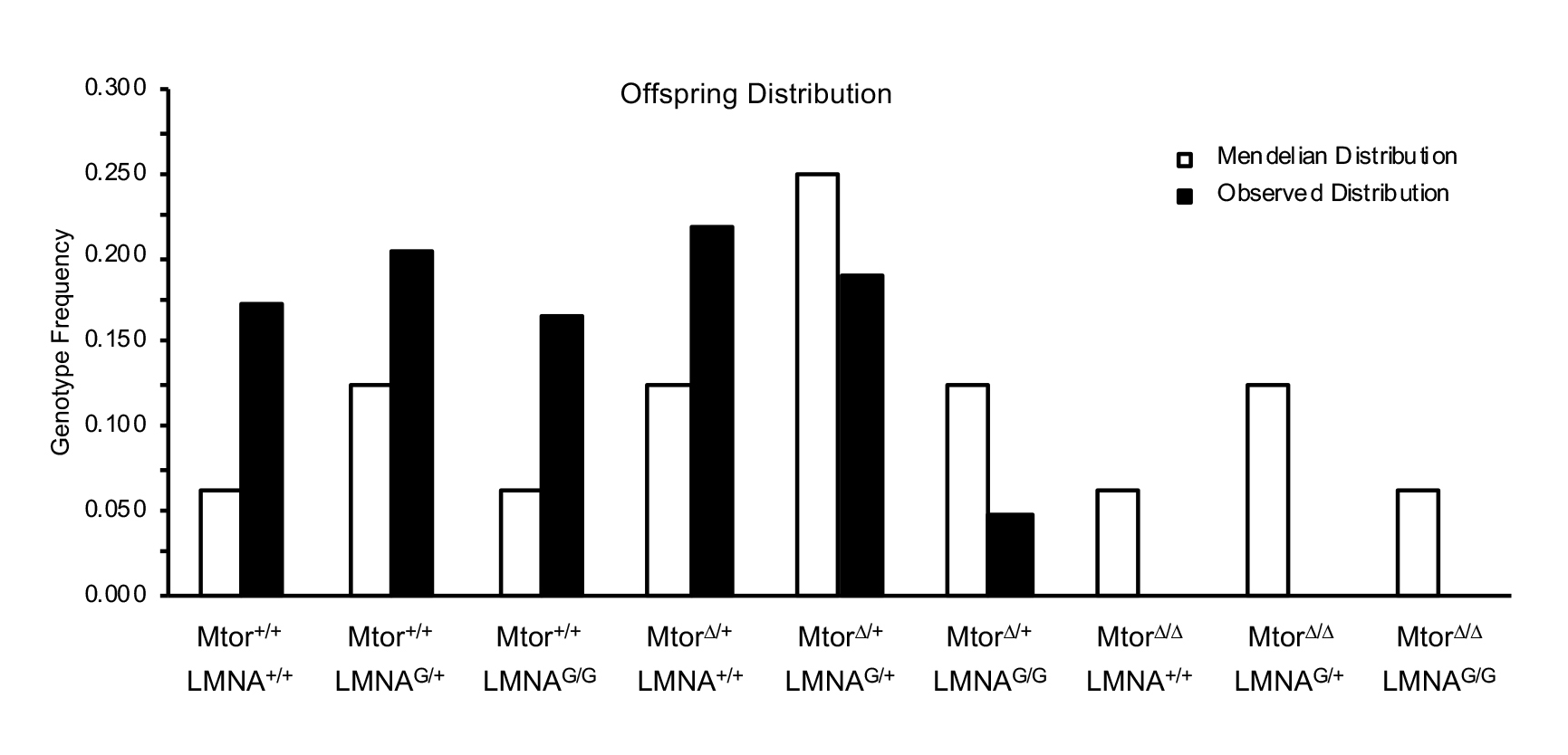
**

**Supplementary Figure 4**

**
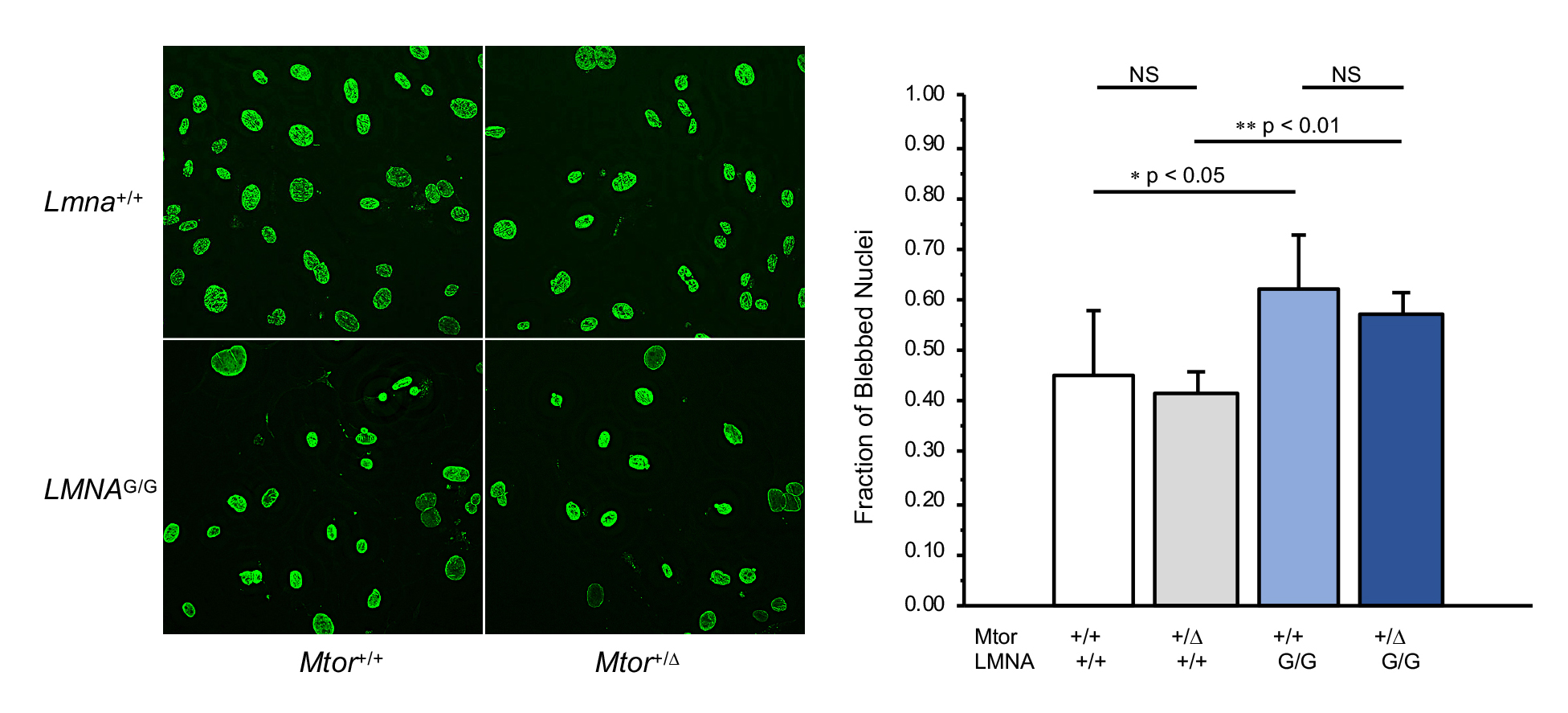
**

**Supplementary Figure 5**

**
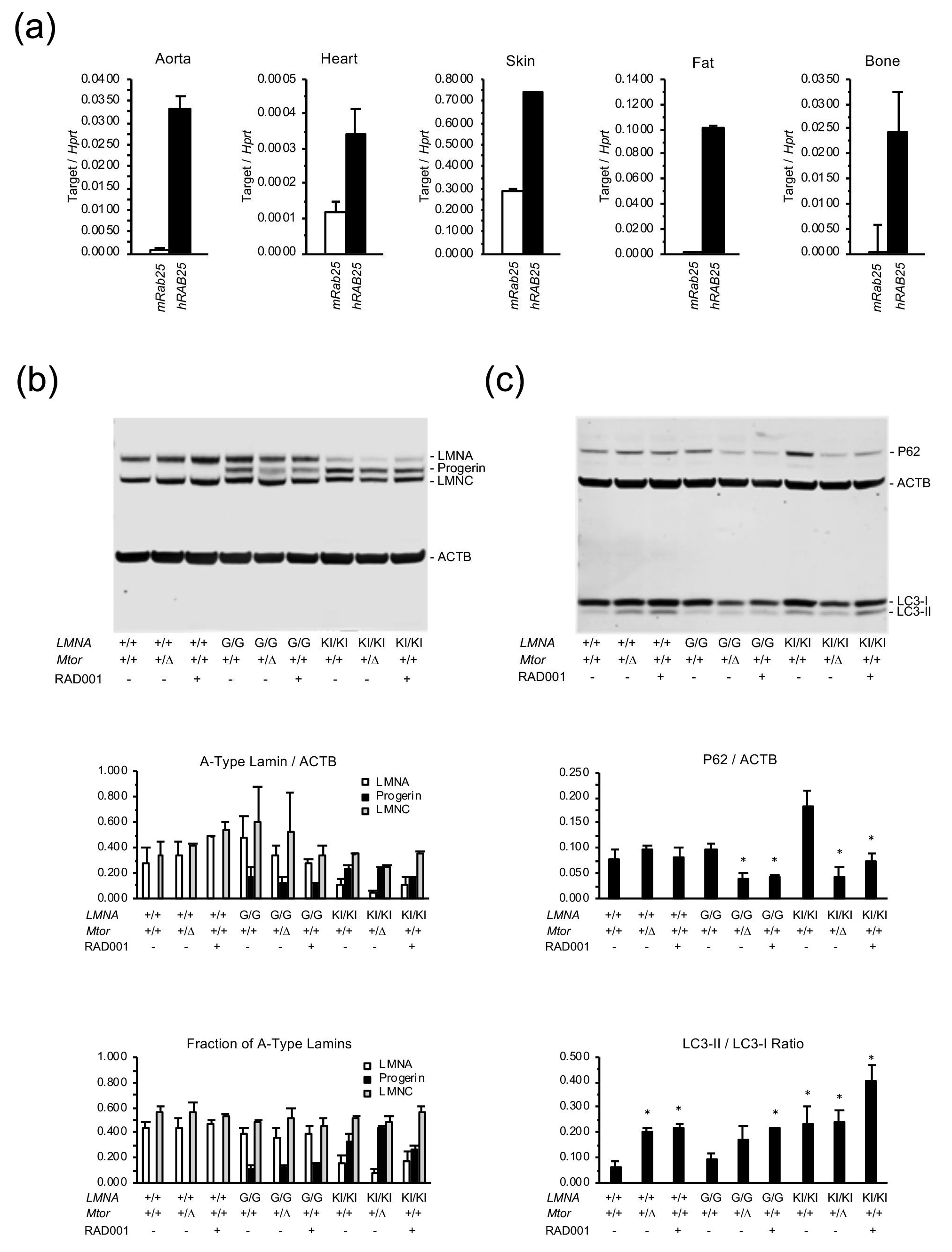
**

**Supplementary Figure 6**

**
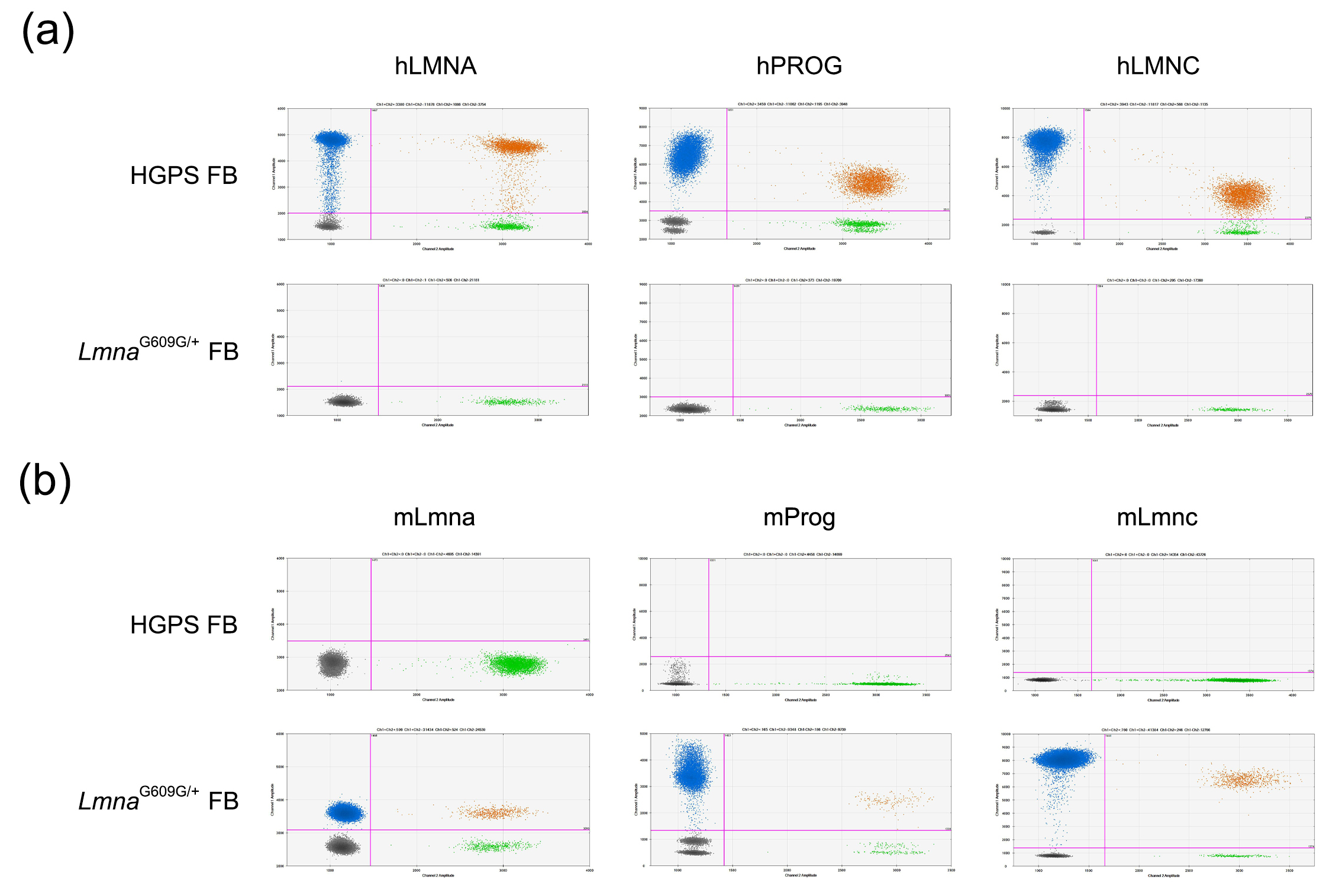
**
